# Fetal bisphenol and phthalate exposure and early childhood growth in a New York City birth cohort

**DOI:** 10.1016/j.envint.2024.108726

**Published:** 2024-05-08

**Authors:** Sophia M. Blaauwendraad, Sarvenaz Shahin, Carol Duh-Leong, Mengling Liu, Kurunthachalam Kannan, Linda G. Kahn, Vincent W.V. Jaddoe, Akhgar Ghassabian, Leonardo Trasande

**Affiliations:** a The Generation R Study Group, Erasmus MC, University Medical Center, Rotterdam, the Netherlands; b Department of Pediatrics, Erasmus MC, University Medical Center, Rotterdam, the Netherlands; c Departments of Pediatrics, New York University Grossman School of Medicine, New York, NY, United States; d Department of Population Health, New York University Grossman School of Medicine, New York, NY, United States; e New York University College of Global Public Health, New York City, NY 10016, United States

## Abstract

**Background:**

Exposure to endocrine-disrupting chemicals such as bisphenols and phthalates during pregnancy may disrupt fetal developmental programming and influence early-life growth. We hypothesized that prenatal bisphenol and phthalate exposure was associated with alterations in adiposity through 4 years. This associations might change over time.

**Methods:**

Among 1091 mother–child pairs in a New York City birth cohort study, we measured maternal urinary concentrations of bisphenols and phthalates at three time points in pregnancy and child weight, height, and triceps and subscapular skinfold thickness at ages 1, 2, 3, and 4 years. We used linear mixed models to assess associations of prenatal individual and grouped bisphenols and phthalates with overall and time-point-specific adiposity outcomes from birth to 4 years.

**Results:**

We observed associations of higher maternal urinary second trimester total bisphenol and bisphenol A concentrations in pregnancy and overall child weight between birth and 4 years only (Beta 0.10 (95 % confidence interval 0.04, 0.16) and 0.07 (0.02, 0.12) standard deviation score (SDS) change in weight per natural log increase in exposure), We reported an interaction of the exposures with time, and analysis showed associations of higher pregnancy-averaged mono-(2-carboxymethyl) phthalate with higher child weight at 3 years (0.14 (0.06, 0.22)), and of higher high-molecular-weight phthalate, di-2-ethylhexyl phthalate, mono-(2-ethyl-5-carboxypentyl) phthalate, mono-(2-carboxymethyl) phthalate, and mono-(2-ethylhexyl) phthalate with higher child weight at 4 years (0.16 (0.04, 0.28), 0.15 (0.03, 0.27), 0.19 (0.07, 0.31), 0.16 (0.07, 0.24), 0.11 (0.03, 0.19)). Higher pregnancy-averaged high-molecular-weight phthalate, di-2-ethylhexyl phthalate, mono-(2-ethyl-5-carboxypentyl) phthalate, mono-(2-ethyl-5-hydroxyhexyl) phthalate, and mono-2(ethyl-5-oxohexyl) phthalate concentrations were associated with higher child BMI at 4 years (0.20 (0.05, 0.35), 0.20 (0.05, 0.35), 0.22 (0.06, 0.37), 0.20 (0.05, 0.34), 0.20 (0.05, 0.34)). For skinfold thicknesses, we observed no associations.

**Discussion:**

This study contributes to the evidence suggesting associations of prenatal exposure to bisphenols and high-molecular-weight phthalates on childhood weight and BMI.

## 1. Introduction

Growth patterns in infancy and early childhood are strong predictors of health in later life. (Leunissen et al., 2009; Chomtho et al., 2008; Ekelund et al., 2007) Rapid postnatal weight gain, particularly in the first year of life, has been shown to significantly increase the odds of childhood obesity. (Zheng et al., 2018) Additionally, alterations in early childhood growth patterns have been associated with adverse vascular, respiratory, and neurodevelopmental outcomes in childhood, as well as higher cardio metabolic risk in later life. (Monasso et al., 2022; Woo, 2019; den Dekker et al., 2016; Sammallahti et al., 2014; Arisaka et al., 2020) Changes in postnatal growth may have their origin in fetal life, as this is a crucial period characterized by high cell turn-over, organ development, and epigenetic programming, which are dependent on endocrine signaling. (Barker, 1990) Exposure to harmful environmental substances, such as endocrine-disrupting chemicals, during pregnancy may disrupt fetal developmental programming and subsequently impact early-life growth.

Phthalates and bisphenols are endocrine-disrupting chemicals whose use has exponentially increased over the past few decades. (World Health O, 2012) Their usage has emerged as an urgent public health concern, as accumulating evidence has demonstrated their detrimental effect on human health. (Kahn et al., 2020) Bisphenols are utilized in the production of polycarbonate resin applied in the lining of metal cans, toys, water pipes, and thermal paper. (Liao and Kannan, 2014; Liao et al., 2012) Low-molecular-weight phthalates are used in personal care products to preserve scent, while high-molecular-weight phthalates are used in vinyl plastics to impart flexibility, pliability, and elasticity. (Serrano et al., 2014; Braun et al., 2013; Vandenberg et al., 2007) Given their widespread application in everyday consumer products, bisphenols and phthalates have become ubiquitous. In pregnant women, bisphenols and phthalates have shown to cross the placental barrier, resulting in direct fetal exposure. (Mose et al., 2007; Nahar et al., 2015) In general, governmental regulations on exposure have been limited to children, such as an embargo of the use of bisphenol A baby bottles, whereas pregnancy women are still frequently exposed.

*In vivo* and *in vitro* studies have demonstrated that bisphenols and phthalates are capable of binding to estrogen, androgen, thyroid, and glucocorticoid receptors, interfering with maternal and fetal hormonal systems. (Vrachnis et al., 2021; Zhang et al., 2019; Acconcia et al., 2015; Van’t Erve et al., 2019) Also, they can bind to the PPAR-gamma (PPARγ) receptor, which plays an important role in placental development and the physiology of pregnancy. (Wieser et al., 2008; Gao et al., 2020) Last, bisphenols and phthalates are shown to cause oxidative stress and have pro-inflammatory capacities. Through these mechanisms, fetal exposure during critical periods of development might disturb later child early-life growth and development.

Previous studies that have assessed associations of prenatal exposure to bisphenols and phthalates with postnatal growth have yielded contradictory results. (Philips et al., 2017; Ferguson et al., 2022; Nidens et al., 2021; Gao et al., 2022) Inconsistencies between those previous studies could be explained by their differences in study populations, small sample sizes, and the number and timing of chemical measurements in pregnancy. Moreover, few studies have considered bisphenols and phthalates that have increasingly been used as replacements for more well-examined chemicals such as bisphenol A (BPA) and di-2-ethylhexyl phthalate (DEHP). Therefore, in the present study, we examined the association of maternal urinary concentrations of bisphenols and phthalates with postnatal adiposity from birth to 4 years among 1091 mother–child pairs.

## 2. Methods

### 2.1. Study design

**Fig. 1.**
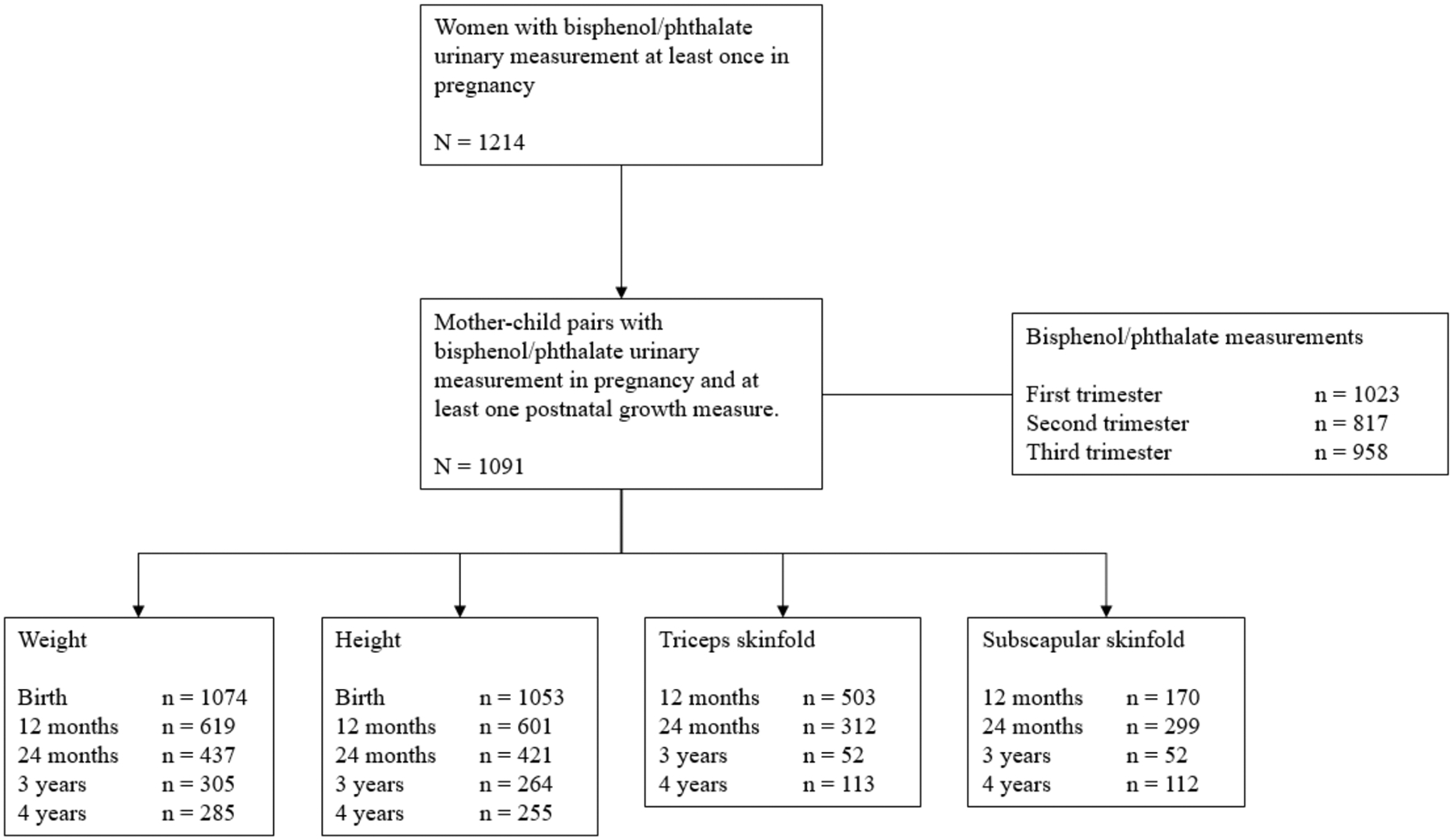
Flowchart of the population.

This study was embedded in the New York University Children’s Health and Environment Study (NYU CHES), an ongoing prospective birth cohort study following mother–child pairs from the prenatal period into childhood. (Trasande et al., 2020) The study was approved by the Institutional Review Board of the NYU Grossman School of Medicine. All participants provided written informed consent at the time of enrollment. Details on the cohort have been previously describe in detail. (Trasande et al., 2020) In brief, between March 2016 and April 2019, NYU CHES recruited participants who were 18 years or older, <18 weeks of gestational age, were fluent in English or Spanish, and planned to delivery at one of 3 NYU-affiliated hospitals: NYU Langone Hospitals – Manhattan, serving a higher income population; NYU Langone – Brooklyn, serving a primarily immigrant community; and Bellevue Hospital, a public hospital serving populations with economic disadvantages. Of the 2193 pregnancies included, a random subsample of 1214 mothers had urinary bisphenol and phthalate metabolite concentrations measured at least once in pregnancy (Fig. 1). Of those, 1091 (89.9 %) mother–child pairs had information on child adiposity up to 4 years.

### 2.2. Urinary bisphenol and phthalate analysis

Maternal spot urine samples were collected during clinical care visits in early (mean 10.8 (standard deviation (SD) 3.2) weeks), mid (mean 20.7 (SD 2.15) weeks), and late (mean 29.2 (SD 3.7) weeks) pregnancy, which could take place at any time during the day. Samples were collected in polyethylene containers, aliquoted into bisphenol- and phthalate-free tubes, and stored at −80 degrees Celsius until chemicals were measured by the NYU Human and Environmental Exposure Analysis Laboratory (HEAL). Once absorbed into the body, phthalates are metabolized into several monoesters that, along with bisphenols, have short half-lives and are excreted primarily in urine. (Wang et al., 2019) Therefore, we consider urinary concentrations as reliable markers of internal human exposure. Phthalate metabolites were measured using enzymatic deconjungation of glucuronidated and sulfated phthalate monoesters followed by solid phase extraction (SPE) coupled with highperformance liquid chromatography electrospray ionization tandem mass spectrometry (HPLC-ESI-MS/MS), as described previously. (Gaylord et al., 2022; Liu et al., 2022) Bisphenols were also measured using HPLC-ESI-MS/MS. (Gaylord et al., 2022) Urinary creatinine was measured by HPLC-MS/MS following a standard protocol as described previously. (Martinez and Kannan, 2018) We measured 22 phthalates metabolites and 8 bisphenols, and included in our analysis any that were detected in at least 50 % of our participants (descriptive statistics of the phthalates and bisphenols are shown in Table S1). Values below the limit of detection (LOD) were imputed with the LOD divided by the square root of 2. (Hornung and Reed, 1990) For each trimester, we grouped phthalates according to their chemical structure into low-molecular-weight (ΣLMW) and high-molecular-weight (ΣHMW) phthalates. We also grouped them according to their parent phthalates into ΣDEHP and di-n-octyl phthalate (ΣDNOP). We grouped all eligible bisphenols into total bisphenol (ΣBP). Details on grouping are described in Text S1. To account for urinary dilution, we corrected for creatinine using a variation of the approach popularized by Boeniger et al. (Boeniger et al., 1993) Chemical concentrations were multiplied by the batch- and time-point-specific median creatinine value divided by the creatinine value of the sample. We used the time-point and batch-specific creatinine values to account for small differences in creatinine between time points and batches arising from non-biological laboratory variation. (Boeniger et al., 1993; Kuiper et al., 2021) To account for right skewedness, bisphenol and phthalate concentrations were natural log transformed. As bisphenols and phthalates are highly variable over time, we calculated the pregnancy-averaged exposure of the individual and grouped creatinine-adjusted metabolites based on one, two or three measurements in pregnancy.

### 2.3. Child growth

As indicators of child growth and adiposity, we included weight, height, and skinfold thicknesses, as those are robust, validated, non-invasive measures of which international growth standards exist. (Pietrobelli et al., 1998; Freedman et al., 2007) Information on birthweight and child sex was obtained from the electronic health records and maternal-report questionnaire if missing. Sex- and gestational age adjusted birth weight z-scores and weight-for-height z-scores were calculated from the International Fetal and Newborn Growth Consortium for the 21st Century standard (INTERGROWTH-21). (Villar et al., 2014) Height, weight, and triceps and subscapular skinfolds were measured during visits which were scheduled for age 1 (eligibility period between 12 and 23 months), 2 (eligibility period between 24 and 35 months), 3–5 years (eligibility period between 36 and 59 months) by trained research assistants at an in-person study visit. Thus, children had either weight measures at 3 years (36 to 48 months) or 4 years (48 to 60 months). A trained examiner measured the child’s weight to the nearest 10 g using a calibrated child scale. Attempts to improve the response rate was to offer evening and weekend visits, offer concierge services to facilitate trips to the research center, and offer to harmonize the research visit with pediatric well visit for children younger than age 2 years. The eligibility age range was by design wide, to improve response rate. Because the wide eligibility age range, we used z-scores to tailor the measurement to an exact age in months. Sex-specific z-scores for age were calculated for weight, height, body mass index (BMI), and skinfolds using World Health Organization growth charts.11 Also, we constructed three categories of infant weight change variables. Infant weight change was defined as change in standard deviation score (SDS) from birth to 2 years (437 of 661 children). If infant weight at 2 years was unavailable, we used weight at 1 year (224 of 661 children). Growth acceleration was defined as more than 0.67 SDS weight increase between time points, normal growth as less than 0.67 SDS increase and less than 0.67 SDS decrease between time points, and growth deceleration as more than 0.67 SDS decrease between time points. A change of 0.67 SDS weight represents the width of each percentile band on standard growth charts, which helps to indicate growth acceleration and deceleration in clinical practice. (Gishti et al., 2014).

### 2.4. Covariates

Information on maternal education (high school or less, college or associate degree, bachelor’s degree or postgraduate degree), race and ethnicity (Hispanic, non-Hispanic White, non-Hispanic Black, non-Hispanic Asian or other/multiple), marital status (married/partnered or single), insurance (public or private), alcohol use (yes or no), smoking during pregnancy (yes or no), and study site (Manhattan, Brooklyn or Bellevue) was collected by questionnaire during prenatal study visits, and missing information was filled in with information from electronic medical records. Information on maternal age at enrollment, pre-pregnancy height and weight, and parity (nulliparous or parous) was obtained from electronic medical records. Maternal height and weight were used to calculate pre-pregnancy BMI.

### 2.5. Statistical analysis

First, we calculated summary statistics for all sociodemographic and lifestyle characteristics of the participants and examined the distribution of continuous covariates through visual inspection of histograms. Summary statistics of normally distributed variables were reported as mean (standard deviation (SD)), of skewed variables as median (95 % range), and of categorical variables as n (%). To identify potential selection bias due to baseline selection or loss-to-follow up, we performed a non-response analysis comparing all women in CHES to our analysis sample. Second, we explored the correlation structure of the exposure data by calculating Spearman correlation coefficients between the exposure groups, within each trimester and between trimesters. We visualized these correlations using heat maps. Third, we used linear mixed-effects models (LME) to estimate the mean difference and 95 % confidence intervals (CI) in SDS of repeated child weight, BMI, triceps skinfold and subscapular skinfold measures for a natural log increase in maternal pregnancy-averaged urinary bisphenol and phthalate measurements. The results of these LME models represent the overall SDS difference in the adiposity outcomes for all measurements between 0 and 4 years per unit increase in maternal urinary chemical concentrations. We applied LME, as those can test the overall associations of exposures with repeated outcome measures and can easily handle missing values. Our model included a random intercept for each participant, as we had repeated measurements of the outcomes per participant, and fixed time points set at birth (for weight only) and 1, 2, 3 and 4 years. To identify which trimester is most important, we ran our models with trimester-specific exposures. Fourth, as preliminary analysis showed a non-linear relation between time and the outcome, we assessed the effect at each study visit (birth, 1, 2, 3, and 4 years) by adding an interaction term between the exposures and postnatal time points to our models. We ran crude and covariate-adjusted models. Confounders were selected from previous literature and defined using a Directed Acyclic Graph (Figure S1). Potential confounders were maternal age, pre-pregnancy BMI, educational level, marital status, insurance, ethnicity, alcohol use, smoking and study site. (Liu et al., 2022) We included those confounders in our models that changed the effect estimates of any of the exposure-outcome combinations for at least 10 %. (Santos et al., 2019) Based on this, all preselected confounders were included in our models.

As a sensitivity analysis, we tested for statistical interaction of child sex with the exposures, considering a p-value threshold of < 0.10, which is a common threshold for identifying interaction. We found some statistically significant interactions and therefore repeated our adjusted LME models for boys and girls separately. Also, as the CHES cohort is an ongoing cohort and we therefore have incomplete data on outcome measurements at 3 and 4 years which could lead to selection bias, we repeated our adjusted LME models including outcomes up to 2 years only. Last, to assess the effect of selection bias due to selective loss-to-follow up, we performed a sensitivity analysis adding inverse probability of censoring weights to our models. (Naimi et al., 2014) We used logistic regression propensity score models to calculate weights for censored or included. The censored group included women that had bisphenol and phthalate measures in pregnancy but no data on child adiposity (*n* = 123). The underlying propensity score models included baseline characteristics potentially related to nonresponse. As loss to follow up bias often arises from socioeconomic inequalities, these characteristics were maternal age, ethnicity, education, marital status, parity, insurance, BMI, alcohol use and smoking in pregnancy. We checked for weight model misspecification by exploring the distributions of the weight, and scaled weights within the population for analysis. The inverse probability weight can be used to address selection bias under the four assumptions of consistency, exchangeability, positivity, and use of the correct model. The consistency hold as previous literature has indeed shown that exposure (the sociodemographic factors) are in general indeed associated with the outcome (the loss to follow up). The positivity assumption holds when at every level of the confounders, there are cases with and without the outcome, which is the case for our included and loss-to-follow-up population. We aimed to fulfill the exchangeability assumption by including many potential influential factors. Last, as the outcome was binary, we chose logistic regression models. (Cole and Hernán, 2008).

Rapid infant weight gain, or a greater than 0.67 increase in weight-for-age z-score between two time points in the first two years of life has most strongly been associated with future cardio metabolic disease. (Zheng et al., 2018) Additionally, within the CHES cohort, oxidative stress, which is a known working mechanism of phthalates and bisphenols, has been proposed as potential mechanism on infant rapid weight gain. (Duh-Leong et al., 2023) Therefore, as a secondary analysis, we assessed the association of maternal pregnancy-averaged urinary bisphenol and phthalate measurements with infant growth patterns, categorized as growth acceleration, normal growth, or growth deceleration. We used logistic regression models to estimate the odds (95 % CI) on infant growth acceleration or deceleration per natural log increase in maternal urinary chemical concentrations, adjusted for the same covariates as our previous covariate-adjusted models.

All statistical tests were 2-sided and p-values for all analysis were presented. To correct for multiple hypothesis testing, we applied False Discovery Rate (FDR) adjustment for each outcome. (Haynes, 2013) We considered an FDR adjusted p-value of < 0.05 statistically significant. Analyses were performed using R Statistical Software (version 4.3.2; R Development Core Team). The percentage missing of covariates was low (0 to 0.5 %). Therefore, we did not perform multiple imputation of missing data.

## 3. Results

### 3.1. Characteristics

Table 1 shows the characteristics of our study population. Mothers had a mean age of 31.8 years at enrollment and median pre-pregnancy BMI of 25.0 kg/m^2^. They were mostly of Hispanic (52.6 %) or Non-Hispanic White (31.1 %) ethnicity, 34.6 % had high school education or less, 88.8 % were married or living with a partner, and 48.8 % were nulliparous. Compared with participants who were excluded due to missing outcome measures, participants included in our analyses were less highly educated, had slightly different race/ethnicities, and more often had public insurance (Table S2). Outcome characteristics are shown in Table 2. The correlations of the exposures within and between trimesters was generally low or very low, except for the correlation of different HMW phthalate metabolites within trimesters, which varied from moderate to very high (Figure S2 and S3).

### 3.2. Pregnancy bisphenols and phthalates and child adiposity

After FDR correction for multiple testing, we observed no effect of pregnancy-averaged bisphenol and phthalate concentration on overall child adiposity outcomes between birth and 4 years (Table 3, Table S3). Trimester-specific analysis showed associations of higher second trimester ΣBP and BPA with higher overall child weight between birth and 4 years (Beta = 0.10 (95 % confidence interval [CI] 0.04, 0.16) and 0.07 (95 % CI 0.02, 0.12) SDS change in weight per natural log increase in exposure, respectively) (Table S4). No trimester-specific associations were present for overall child BMI or skinfold thicknesses (Table S5–S7).

**Fig. 2.**
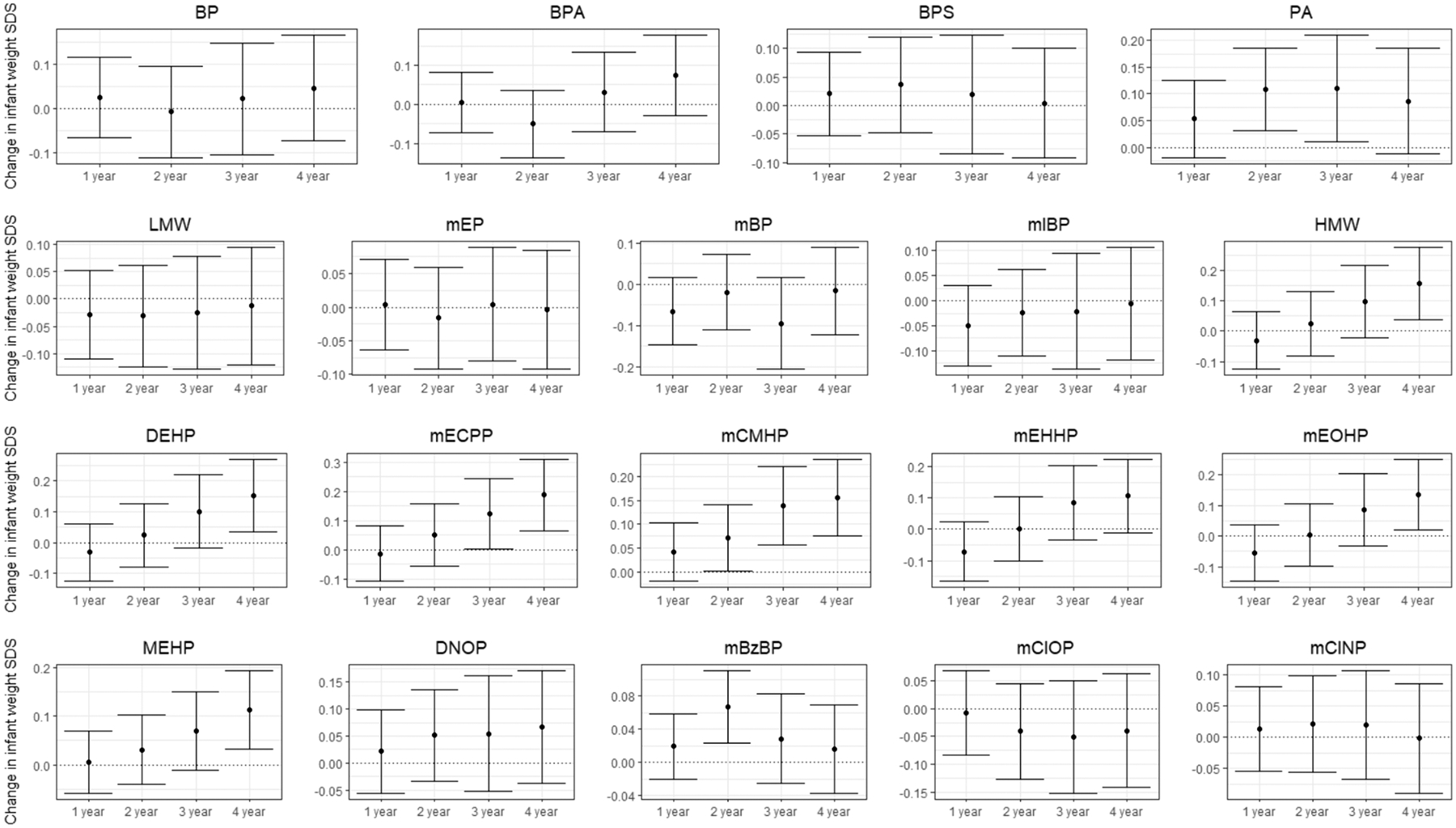
Effect of exposures on child weight at each time point, adjusted model (n = 1091). Values are derived from linear mixed models and represent the standard deviation score (SDS) change in weight (95 % confidence interval) per natural log increase in the chemical exposure for each time point. Weight SDS are sex-specific z-scores for age according to World Health Organization growth charts. Model is adjusted for race, education, marital status, insurance, parity, body mass index, alcohol and tabacco use. BP total bipshenol; BPA bisphenol A; BPS bisphenol S; PA phthalic acid; LMW low molecular weight phthalate; mEP mono-ethyl phthalate; mnBP mono-n-butyl phthalate; mIBP mono-isobutyl phthalate; HMW high molecular weight phthalate; DEHP di-(2-ethylhexyl) phthalate; mECPP mono-(2-ethyl-5-carboxypentyl) phthalate; mCMHP mono-(2-carboxymethyl) phthalate; mEHHP mono-(2-ethyl-5-hydroxyhexyl) phthalate; mEOHP mono-2(ethyl-5-oxohexyl) phthalate; mEHP mono-(2-ethylhexyl) phthalate; DNOP di-*n*-octyl phthalate; mCPP mono-(3-carboxypropyl) phthalate; mBzP mono-benzyl phthalate; mCIOP mono-(carboxyisooctyl) phthalate; mCINP mono-(carboxyisononyl) phthalate. Corresponding data are shown in Table S8.

**Fig. 3.**
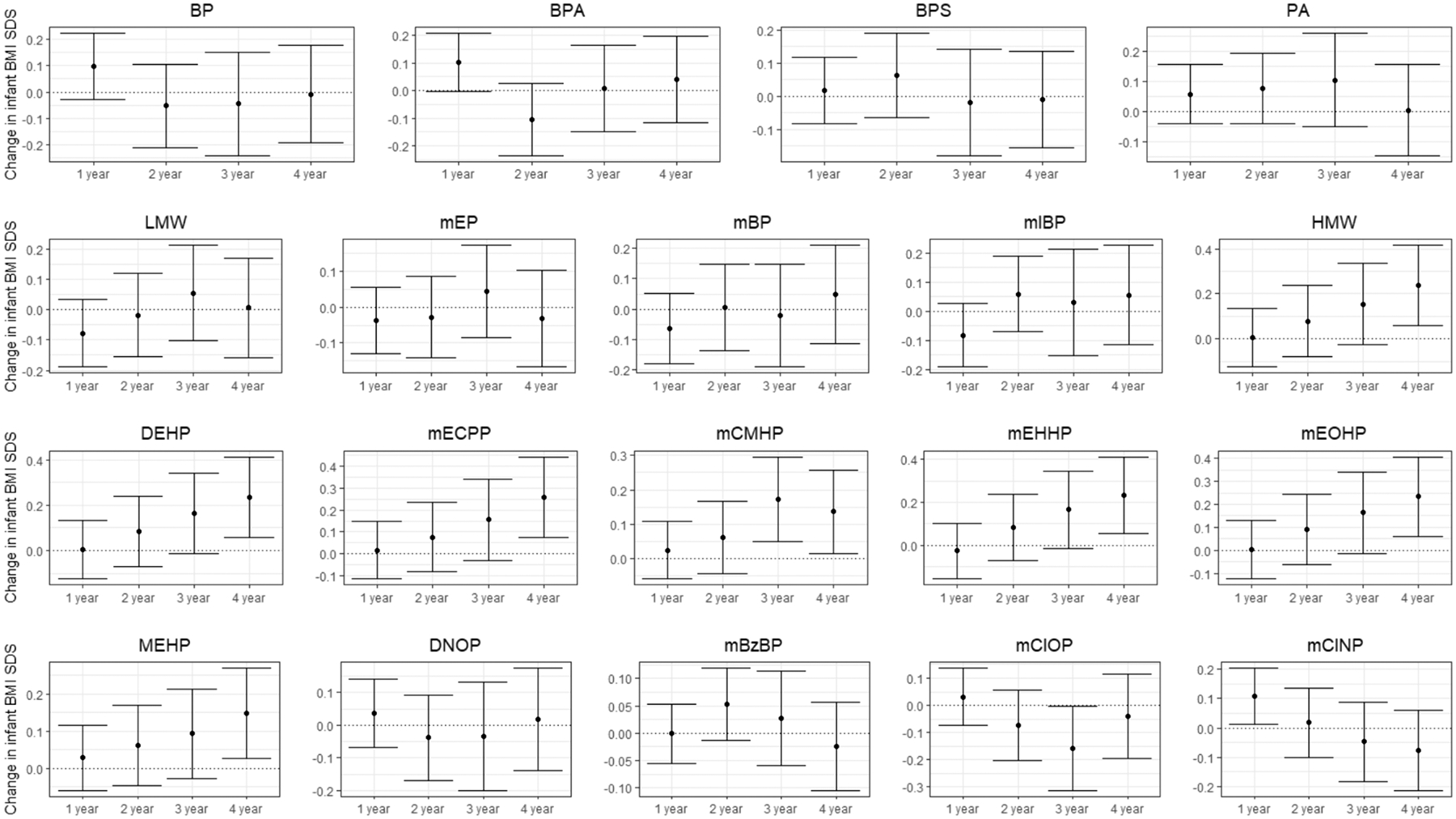
Effect of exposures on child body mass index at each time point, adjusted model (n = 672). Values are derived from linear mixed models and represent the SDs change in weight (95 % confidence interval) per natural log increase in the chemical exposure for each time point. Body mass index SDs are sex-specific z-scores for age according to World Health Organization growth charts. Model is adjusted for race, education, marital status, insurance, parity, body mass index, alcohol and tabacco use. BP total bipshenol; BPA bisphenol A; BPS bisphenol S; PA phthalic acid; LMW low molecular weight phthalate; mEP mono-ethyl phthalate; mnBP mono-n-butyl phthalate; mIBP mono-isobutyl phthalate; HMW high molecular weight phthalate; DEHP di-(2-ethylhexyl) phthalate; mECPP mono-(2-ethyl-5-carboxypentyl) phthalate; mCMHP mono-(2-carboxymethyl) phthalate; mEHHP mono-(2-ethyl-5-hydroxyhexyl) phthalate; mEOHP mono-2(ethyl-5-oxohexyl) phthalate; mEHP mono-(2-ethylhexyl) phthalate; DNOP di-*n*-octyl phthalate; mCPP mono-(3-carboxypropyl) phthalate; mBzP mono-benzyl phthalate; mCIOP mono-(carboxyisooctyl) phthalate; mCINP mono-(carboxyisononyl) phthalate. Corresponding data are shown in Table S9.

Analysis of exposures with child outcome at each separate time point showed associations of higher pregnancy-averaged mono-(2-carboxymethyl) phthalate (mCMHP) with higher child weight at 3 years (0.14 (95 % CI 0.06, 0.22) SD change in weight per natural log increase in mCMHP), and of higher ΣHMW, ΣDEHP, mono-(2-ethyl-5-carboxypentyl) phthalate (mECPP), mCMHP, and mono-(2-ethylhexyl) phthalate (MEHP) with higher child weight at 4 years (ΣHMW: 0.16 (95 % CI 0.04, 0.28), ΣDEHP: 0.15 (95 % CI 0.03, 0.27), mECPP: 0.19 (95 % CI 0.07, 0.31), mCMHP: 0.16 (95 % CI 0.07, 0.24), MEHP: 0.11 (95 % CI 0.03, 0.19)) (Fig. 2, Table S8). Likewise, higher pregnancy-averaged ΣHMW, ΣDEHP, mECPP, mEHHP, and mono-2(ethyl-5-oxohexyl) phthalate (mEOHP) concentrations were associated with higher child BMI at 4 years (ΣHMW: 0.20 (95 % CI 0.05, 0.35), ΣDEHP: 0.20 (95 % CI 0.05, 0.35), mECPP: 0.22 (95 % CI 0.06, 0.37), mEHHP: 0.20 (95 % CI 0.05, 0.34), mEOHP: 0.20 (95 % CI 0.05, 0.34) SD change in BMI per natural log increase in exposure) (Fig. 3, Table S9). For child skinfold thickness, we observed no associations with separate time points (Table S10–11).

### 3.3. Sensitivity analysis

In our sex-specific analysis, all associations of the exposure with overall adiposity outcomes between birth and 4 years remained non-significant for boys and girls (Table S12–13). Repeating our adjusted LME model including outcomes up to 2 years only yielded similar results as the model including outcomes up to 4 years (Table S14). Last, adding inverse probability of censoring weight to our model on the overall association with adiposity outcomes between birth and 4 years did not change our effect estimates (Table S15).

### 3.4. Infant growth patterns

In our multivariable logistic regression models, no significant associations of maternal pregnancy-averaged bisphenol or phthalate urinary concentrations with infant growth deceleration or acceleration were present (Table S16).

## 4. Discussion

In our prospective birth cohort study in New York City, we observed positive associations of maternal urinary second trimester ΣBP and BPA concentrations in pregnancy and overall child weight between birth and 4 years only. Also, we reported an interaction of the exposure with time, and analysis of specific study visits showed associations of higher ΣHMW phthalates with higher child weight and body mass index at 3 and 4 years of age. We observed no consistent associations of bisphenols and phthalates with child skinfold thicknesses between age 1 and 4 years.

### 4.1. Interpretation of main findings

Previous studies have investigated associations of prenatal exposure to bisphenols and phthalates with child weight and BMI. Some, but not all, have reported positive associations of prenatal exposure to the LMW phthalates mEP and mBP with child weight or BMI. (Ferguson et al., 2022; Botton et al., 2016)Also, for HMW phthalates, higher prenatal exposure to mECPP, mEHHP, mBzP, mCPP, and mCIOP have been associated with child adiposity outcomes. (Ferguson et al., 2022; Gao et al., 2022; Yang et al., 2018) In some studies, those associations were sex- and trimester specific. In contrast, in a German cohort, no associations were reported of 13 phthalates measured in prenatal spot urine samples with child weight up to 2 years. (Nidens et al., 2021) Interestingly, recently an US cohort investigating the association of prenatal phthalate exposure on child weight at specific time points from pregnancy until child age 6 years reported associations at 3 and 4 years only. (Ferguson et al., 2022) In the current study, we also observed associations of higher pregnancy-averaged exposure to the ΣHMW and the individual metabolites mCMHP, mECPP, and MEHP with higher weight and BMI at 3 and 4 years.

On bisphenols, studies are more inconsistent. A Greek study reported associations of higher first trimester BPA with lower adiposity measures in girls, but higher adiposity measures in boys between age 6 months and 4 years. (Vafeiadi et al., 2016) A Spanish study reported associations of higher BPA with higher waist circumference and BMI at age 4 years, but not at 6 or 14 months. (Valvi et al., 2013) Three other studies from France, the US, and Mexico reported no associations of prenatal BPA with child weight or BMI. (Yang et al., 2018; Philippat et al., 2014; Braun et al., 2014) In the current study, we observed an effect of prenatal exposure to total bisphenols, and in particularly BPA, in second trimester with higher overall child weight up to 4 years.

Comparing urinary concentrations of phthalates and bisphenols of our study sample to other studies on prenatal chemical exposure and early-life growth showed that we had phthalate concentration comparable to another US cohort (Ferguson et al., 2022), except for lower concentrations of DINCH metabolites. This cohort indeed detected associations of prenatal CIOP with higher weight at 4 years, thus our lack of association might be explained by lower concentrations. Two studies from Mexico City (Yang et al., 2018) and France (Botton et al., 2016) had higher concentrations of LMW and HMW as compared to our cohort, a study from China (Gao et al., 2022) had different concentration depending on the phthalate, and a German (Nidens et al., 2021) cohort had lower concentrations of HMW. These data show that exposure is highly dependent on geographical area and characteristics of the study population. To overcome these differences, future research should focus on individual participant data *meta*-analysis including cohorts from different countries.

We hypothesize several mechanisms through which bisphenols and phthalates might interfere with fetal lipid metabolism. Bisphenols and phthalates have been shown to bind to the nuclear transcription factors PPAR gamma (PPARγ) and its heterodimer retinoid X receptor (RXR), which are expressed predominantly in adipose tissue and act as regulators of adipocyte differentiation and lipid metabolism. (Philips et al., 2017) PPARγ agonists have shown to cause lipid accumulation and release of adipocyte-related hormones, resulting in an increased susceptibility to obesity. (Philips et al., 2017) Bisphenols, and to a lesser extent phthalates, have pro-estrogenic capacities. Estrogens are a critical determinant of body fat metabolism, and thus bisphenols might directly influence body fat development *in utero* or indirectly, and potentially more chronically, through disturbance of the development of the endocrine axis. (Steiner and Berry, 2022) Also, bisphenol and phthalate exposure in pregnancy might disturb maternal and neonatal thyroid hormone levels. (Derakhshan et al., 2021; Derakhshan et al., 2021) Thyroid hormones have prominent effects on hepatic fatty acid and cholesterol synthesis and metabolism, and maternal thyroid hormone levels in turn have been shown to influence child fat distribution. (Sinha et al., 2018; Godoy et al., 2014) Lastly, bisphenols and phthalates, in particular BPA and MEHP, are capable of binding to glucocorticoid receptors, which regulate metabolic adaptation during stress, and chronic increases in glucocorticoid have been associated with metabolic syndrome. (Lee et al., 2018).

Adipose tissue in humans mainly develops from the second trimester onward, as primitive fat lobules start to develop at 14 weeks of gestational age and increase in number until approximately 23 weeks. (Desoye and Herrera, 2021; Poissonnet et al., 1983) Thereafter, further development of the adipocytes and capillary network takes place. The association of prenatal bisphenol exposure with overall childhood BMI was present after second trimester exposure, which might be explained by the developmental stage of the fat lobules. For the HMW phthalates, associations of the exposures were only present with weight and BMI at 3 and 4 years. Potentially prenatal phthalate exposure increases child susceptibility to adiposity, for instance through PPARγ agonism. It is possible that this susceptibility manifests after infancy, when children no longer receive breast feeding and have been introduced to a Western diet.

Our study adds to a body of evidence and has an important contribution in enlightening the effects of prenatal exposure to bisphenols and phthalates on child adiposity outcomes. As compared to previous studies, our study sample was the largest. (Ferguson et al., 2022; Nidens et al., 2021; Philippat et al., 2014; Braun et al., 2014; Valvi et al., 2013) Additionally, we are among the first to include newly introduced chemicals of whom the production is rapidly rising, such as DINCH and DINP. (Ferguson et al., 2022) Next to our study, only one study included three urinary measures in pregnancy, which not only enabled accounting for variability of the chemicals, but also identification of windows of high susceptibility throughout pregnancy. (Gao et al., 2022).

Child adiposity has been associated with, and might potentially mediate, an adverse cardio metabolic profile in childhood, subsequently increasing the odds of cardio metabolic disease in later life. To gain more insight on the adverse effects of prenatal bisphenol and phthalate exposure, the heterogeneity of previous evidence requires future multi-cohort studies including multiple diverse population, using repeated measurements of bisphenols and phthalates in pregnancy,

### 4.2. Methodological considerations

The current study benefitted from prospective data collection from early pregnancy onward in a large population, which enabled repeated measurements of bisphenols and phthalates throughout pregnancy and collection of a wide range of covariates. Our population was diverse with respect to race, ethnicity, income, and education, which promotes the generalizability of our results. Also, we had repeated measures of child adiposity, allowing us to use LME. A limitation of the bisphenol and phthalate measurements is that they are highly variable over time and excreted from the body within 24 h. (Mattison et al., 2014; Hauser et al., 2004) The trimester-specific analysis might have been sensitive to measurement errors, as we had to rely on one measurement for each trimester. (Pollack et al., 2016) However, previous studies have shown that urinary phthalate measurements might reasonably reflect exposures in the prior several weeks or even months. (Townsend et al., 2013) We observed no associations of maternal bisphenol and phthalate urinary concentrations with skinfold thicknesses. However, as our sample size of skinfold measures was small, especially at age 3 and 4 years, our results on skinfold thicknesses should be interpreted with caution. Early-life growth and adiposity are dependent on a lot of factors. However, these factors will only influence the studied associations if they are both associated with the exposure and the outcome. Based on literature, we aimed to identify and adjust for such factors. However, although we adjusted for many potential confounders, residual confounding due to the observational nature of the study might have occurred. Last, the loss to follow-up from birth to 4 years could have caused selection bias. We aimed to take this into account by including inverse probability of censoring weight in a sensitivity analysis.

## 5. Conclusion

In our population-based prospective cohort study, we observed positive associations of maternal second trimester urinary ΣBP and BPA concentrations in pregnancy and child weight between birth and 4 years, and of pregnancy-averaged concentrations of ΣHMW and several HMW phthalate metabolites with child weight and BMI at 3 and 4 years of age. Our study adds to a body of evidence on the effects of prenatal bisphenol and phthalate exposure. Future multi-cohort studies including multiple diverse population, using repeated measurements of bisphenols and phthalates in pregnancy and of child adiposity outcomes are needed.

## Supplementary Material

Supplementary Materials

## Figures and Tables

**Table 1:** General characteristics of the analytic sample.

	N = 1091
**Maternal characteristics**	
Age in years, mean (±SD)	31.8 (5.6)
Highest education finished, n (%)	
High school or less	375 (34.6)
College	117 (10.8)
Associate degree	67 (6.2)
Bachelor’s degree	245 (22.6)
Postgraduate degree	281 (25.9)
Race/ethnicity, n (%)	
Hispanic	572 (52.6)
Non-Hispanic White	340 (31.3)
Non-Hispanic Black	58 (5.3)
Non-Hispanic Asian	88 (8.1)
Other	10 (0.9)
Multiple	19 (1.7)
Marital status, n (%)	
Married/living with a partner	969 (88.8)
Divorced/separated	18 (1.6)
Single/widowed	104 (9.5)
Insured, n (%)	
Public	591 (54.5)
Private	494 (45.5)
Parity, n (%)	
Nullipara	552 (48.8)
Multipara	580 (51.2)
Pre-pregnancy body mass index in kg/m^2^, median (95 % range)	25.0 (18.7, 41.8)
Alcohol use, n (%)	
Never	376 (34.6)
Stopped in pregnancy	550 (50.6)
Continued using alcohol	160 (14.7)
Smoking, n (%)	
Never	991 (91.1)
Stopped in pregnancy	79 (7.2)
Continued in pregnancy	18 (1.7)
Hospital of recruitment	
Bellevue Hospital	283 (25.9)
NYU Brooklyn	299 (27.4)
NYU Manhattan	509 (46.7)

Values presented as mean (±standard deviation (SD), median (95 % range) or number of participants (valid %). Number of missing values per covariate, *n* (%): ethnicity 4 (0.4), education 6 (0.5), pre-pregnancy body mass index 7 (0.6), alcohol use 5 (0.5), smoking 3 (0.3).

**Table 2:** Outcome characteristics of the study population.

	N = 1091
Child sex, n (%)	
Male	612 (52.9)
Female	544 (47.1)
Gestational age at birth, weeks, median (95 % range)	39.3 (34.8, 41.3)
Birth weight, kg, mean (±SD)	3.3 (0.5)
Birth length, cm, mean (±SD)	50.4 (2.7)
*1-year visit*	
Age, months, median (95 % range)	12.6 (11.2, 20.8)
Weight, kg, mean (±SD)	10.3 (1.4)
Height, cm, mean (±SD)	76.5 (3.7)
Triceps skinfold thickness, mm, median (95 % range)	11.4 (7.1, 20.6)
Subscapular skinfold thickness, mm, median (95 % range)	7.7 (5.3, 12.1)
*2-year visit*	
Age, months, median (95 % range)	25.2 (23.2, 35.5)
Weight, kg, mean (±SD)	13.6 (2.3)
Height, cm, mean (±SD)	88.9 (5.0)
Triceps skinfold thickness, mm, median (95 % range)	11.0 (6.3, 18.0)
Subscapular skinfold thickness, mm, median (95 % range)	7.1 (4.7, 13.0)
*3-year visit*	
Age, months, median (95 % range)	39.5 (36.1, 47.5)
Weight, kg, median (95 % range)	16.3 (12.6, 25.7)
Height, cm, mean (±SD)	98.7 (5.0)
Triceps skinfold thickness, mm, median (95 % range)	12.2 (7.0, 21.3)
Subscapular skinfold thickness, mm, median (95 % range)	7.2 (5.2, 15.1)
*4-year visit*	
Age, months, median (95 % range)	50.5 (48.1, 59.4)
Weight, kg, median (95 % range)	18.3 (14.2, 32.3)
Height, cm, mean (±SD)	105.4 (5.1)
Triceps skinfold thickness, mm, median (95 % range)	12.5 (7.6, 24.8)
Subscapular skinfold thickness, mm, median (95 % range)	7.6 (4.8, 22.7)
*Growth patterns 0 to 2 years*	
Growth deceleration	104 (16.1)
Normal growth	300 (46.4)
Growth acceleration	242 (37.5)

Values presented as mean (±standard deviation (SD)) or median (95 % range).

**Table 3:** Associations of the individual and grouped metabolites with overall child adiposity outcomes between birth and 4 years, adjusted model.

	Weight in SDS (n = 1091)			Body mass index in SDS (n = 672)			Triceps skinfold in SDS (n = 558)			Subscapular skinfold in SDS (n = 415)		
Chemical	Estimate (95 % CI)	Nominal p-value	FDR P-value	Estimate (95 % CI)	Nominal p-value	FDR P-value	Estimate (95 % CI)	Nominal p-value	FDR P-value	Estimate (95 % CI)	Nominal p-value	FDR P-value
BP	0.06 (0.00, 0.13)*	0.047	0.259	0.07 (−0.03, 0.17)	0.151	0.275	0.05 (−0.07, 0.14)	0.423	0.478	−0.06 (−0.18, 0.07)	0.353	0.542
BPA	0.06 (0.01, 0.12)*	0.021	0.259	0.08 (0.00, 0.17)	0.057	0.182	0.02 (−0.07, 0.12)	0.605	0.637	0.01 (−0.09, 0.11)	0.801	0.942
BPS	0.03 (−0.02, 0.08)	0.225	0.375	0.03 (−0.05, 0.11)	0.473	0.592	0.04 (−0.05, 0.14)	0.430	0.478	−0.08 (−0.18, 0.02)	0.136	0.432
PA	0.05 (0.00, 0.09)	0.054	0.259	0.10 (0.02, 0.18)*	0.019	0.095	−0.05 (−0.14, 0.04)	0.235	0.356	−0.08 (−0.18, 0.01)	0.080	0.400
LMW	0.00 (−0.06, 0.05)	0.954	0.994	−0.07 (−0.16, 0.02)	0.112	0.224	−0.06 (−0.16, 0.04)	0.236	0.356	−0.11 (−0.21, 0.00)	0.058	0.400
mEP	0.00 (−0.05, 0.05)	0.994	0.994	−0.04 (−0.11, 0.03)	0.269	0.359	−0.05 (−0.13, 0.04)	0.235	0.356	−0.09 (−0.18, 0.00)	0.046*	0.400
mBP	−0.01 (−0.07, 0.05)	0.776	0.913	−0.06 (−0.15, 0.03)	0.226	0.348	−0.07 (−0.17, 0.03)	0.198	0.356	−0.03 (−0.14, 0.08)	0.581	0.829
mIBP	0.00 (−0.06, 0.05)	0.977	0.994	−0.05 (−0.14, 0.04)	0.253	0.359	0.01 (−0.08, 0.10)	0.782	0.782	0.01 (−0.09, 0.11)	0.912	0.973
HMW	0.05 (−0.01, 0.12)	0.126	0.259	0.09 (−0.01, 0.19)	0.089	0.198	0.09 (−0.02, 0.17)	0.127	0.356	0.08 (−0.05, 0.21)	0.224	0.432
DEHP	0.05 (−0.01, 0.12)	0.117	0.259	0.09 (−0.01, 0.20)	0.073	0.182	0.09 (−0.02, 0.17)	0.128	0.356	0.08 (−0.04, 0.21)	0.207	0.432
mECPP	0.05 (−0.01, 0.12)	0.130	0.259	0.10 (0.00, 0.21)	0.059	0.182	0.11 (−0.01, 0.19)	0.069	0.356	0.12 (−0.01, 0.25)	0.076	0.400
mCMHP	0.05 (0.01, 0.09)*	0.027	0.259	0.09 (0.02, 0.16)*	0.011	0.095	0.04 (−0.03, 0.15)	0.249	0.356	0.06 (−0.03, 0.14)	0.203	0.432
mEHHP	0.06 (−0.01, 0.12)	0.081	0.259	0.07 (−0.04, 0.17)	0.216	0.348	0.07 (−0.04, 0.15)	0.225	0.356	0.08 (−0.05, 0.20)	0.238	0.432
mEOHP	0.06 (−0.01, 0.12)	0.085	0.259	0.09 (−0.01, 0.19)	0.068	0.182	0.09 (−0.02, 0.17)	0.123	0.356	0.08 (−0.05, 0.21)	0.223	0.432
MEHP	0.03 (−0.01, 0.08)	0.165	0.299	0.09 (0.02, 0.16)*	0.017	0.095	0.05 (−0.03, 0.16)	0.222	0.356	0.05 (−0.03, 0.14)	0.254	0.432
DNOP	0.02 (−0.03, 0.08)	0.365	0.522	0.02 (−0.06, 0.11)	0.595	0.626	0.04 (−0.05, 0.15)	0.366	0.458	0.00 (−0.10, 0.11)	0.973	0.973
mCPP	0.02 (−0.03, 0.08)	0.365	0.522	0.02 (−0.06, 0.11)	0.595	0.626	0.04 (−0.05, 0.15)	0.366	0.458	0.00 (−0.10, 0.11)	0.973	0.973
mBzP	−0.01 (−0.03, 0.02)	0.625	0.781	0.01 (−0.03, 0.06)	0.523	0.615	−0.04 (−0.09, 0.02)	0.132	0.356	−0.01 (−0.06, 0.05)	0.790	0.942
mCIOP	0.02 (−0.03, 0.07)	0.483	0.644	−0.02 (−0.10, 0.07)	0.681	0.681	0.07 (−0.02, 0.17)	0.128	0.356	0.06 (−0.04, 0.16)	0.259	0.432
mCINP	0.04 (−0.01, 0.08)	0.126	0.259	0.09 (0.02, 0.17)*	0.017	0.095	0.05 (−0.03, 0.16)	0.239	0.356	−0.02 (−0.11, 0.07)	0.695	0.926

Values are obtained from linear mixed models and represent the standard deviation score (SDS) change in overall adiposity outcomes (95 % confidence interval (CI)) between birth and 4 years per natural log increase in the chemical exposure. Outcome SDS are sex-specific z-scores for age according to World Health Organization growth charts. Model is adjusted for race, education, marital status, insurance, parity, body mass index, alcohol and tabacco use. BP total bipshenol; BPA bisphenol A; BPS bisphenol S; PA phthalic acid; LMW low molecular weight phthalate; mEP mono-ethyl phthalate; mnBP mono-n-butyl phthalate; mIBP mono-isobutyl phthalate; HMW high molecular weight phthalate; DEHP di-(2-ethylhexyl) phthalate; mECPP mono-(2-ethyl-5-carboxypentyl) phthalate; mCMHP mono-(2-carboxymethyl) phthalate; mEHHP mono-(2-ethyl-5-hydroxyhexyl) phthalate; mEOHP mono-2(ethyl-5-oxohexyl) phthalate; mEHP mono-(2-ethylhexyl) phthalate; DNOP di-*n*-octyl phthalate; mCPP mono-(3-carboxypropyl) phthalate; mBzP mono-benzyl phthalate; mCIOP mono-(carboxyisooctyl) phthalate; mCINP mono-(carboxyisononyl) phthalate.

FDR False Discovery Rate *Nominal adjusted p-value < 0.05.

## Data Availability

Data will be made available on request.
