# Supplementary material for: Fetal bisphenol and phthalate exposure and early childhood growth in a New York City birth cohort
Source: Environ Int. Author manuscript; Available in PMC 2025 Apr 29. (PMC12039796; doi:10.1016/j.envint.2024.108726)
Supplement: Supplementary Materials [file NIHMS2074017-supplement-Supplementary_Materials.docx]

**Supplementary Material**

**Fetal bisphenol and phthalate exposure and early childhood growth in a New York City birth cohort**

**Figure S1.** Directed Acyclic Graph.

**Figure S2.** Correlation plot of the chemical exposures within and between trimesters.

**Text S1.** Details on grouping of the maternal urinary bisphenols and phthalates.

**Table S1.** Values below the limits of detection per chemical

**Table S2.** Non-response analysis of the general characteristics of the study population

**Table S3.** Associations of the individual and grouped metabolites with early childhood adiposity outcomes, basic model

**Table S4.** Associations of the individual and grouped trimester-specific metabolites with early childhood weight between birth and 4 years, adjusted model

**Table S5.** Associations of the individual and grouped trimester-specific metabolites with early childhood body mass index between 1 and 4 years, adjusted model

**Table S6.** Associations of the individual and grouped trimester-specific metabolites with early childhood triceps skinfold between 1 and 4 years, adjusted model

**Table S7.** Associations of the individual and grouped trimester-specific metabolites with early childhood subscapular skinfold between 1 and 4 years, adjusted model

**Table S8.** Effect of exposures on early childhood weight at each time point, adjusted model

**Table S9.** Effect of exposures on early childhood body mass index at each time point, adjusted model

**Table S10.** Effect of exposures on early childhood triceps skinfold at each time point, adjusted model

**Table S11.** Effect of exposures on early childhood subscapular skinfold at each time point, adjusted model

**Table S12.** Associations of the individual and grouped metabolites with childhood adiposity outcomes between birth and 4 years in boys, adjusted model

**Table S13.** Associations of the individual and grouped metabolites with childhood adiposity outcomes between birth and 4 years in girls, adjusted model

**Table S14.** Associations of the individual and grouped metabolites with childhood adiposity outcomes between birth and 2 years, adjusted model

**Table S15.** Associations of the individual and grouped metabolites with childhood adiposity outcomes between birth and 4 years including inverse probability of censoring weights, adjusted model

**Table S16.** Associations of the individual and grouped pregnancy-averaged metabolites with infant growth patterns, adjusted model

**Figure S1.** Directed Acyclic Graph


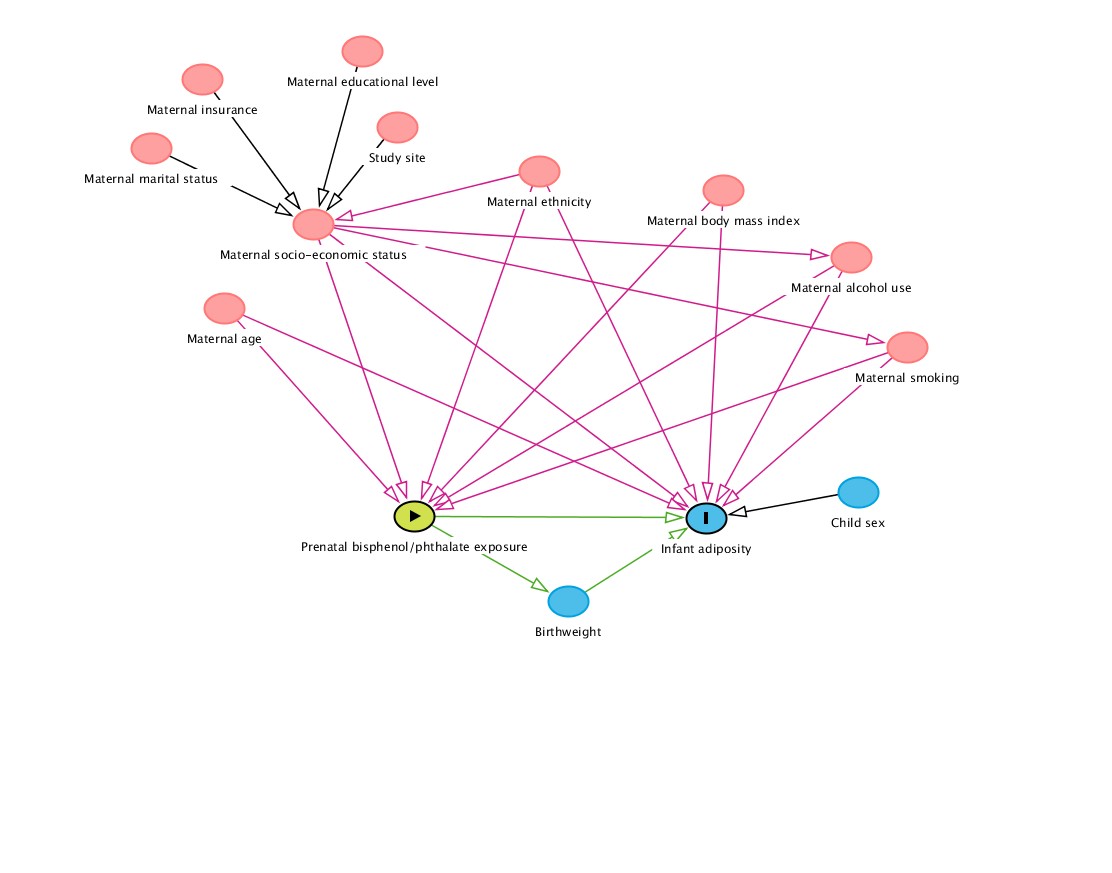


**Figure S2.** Correlation plot of the creatinine-adjusted chemical exposures within and between trimesters. BPA bisphenol A, BPS bisphenol S, cr creatinine adjusted, T1 trimester 1, T2 trimester 2, T3 trimester 3.


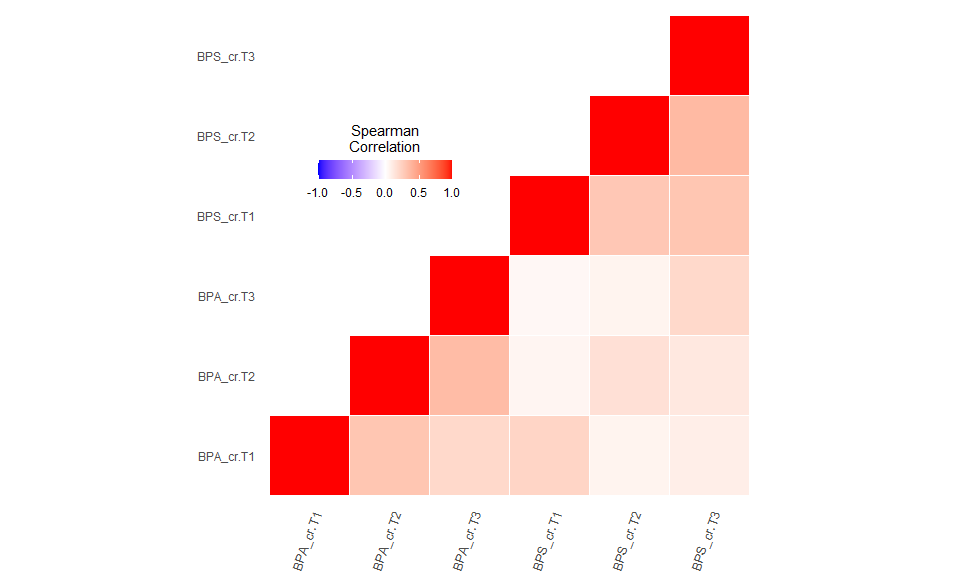


**Figure S3.** Correlation plot of the creatinine-adjusted chemical exposures within and between trimesters. mEP mono-ethyl phthalate; mCPP mono-(3-carboxypropyl) phthalate; PA phthalic acid; mBzP mono-benzyl phthalate; mCIOP mono-(carboxyisooctyl) phthalate; mCINP mono-(carboxyisononyl) phthalate; mBP mono-n-butyl phthalate; mIBP mono-isobutyl phthalate; mECPP mono-(2-ethyl-5-carboxypentyl) phthalate; mCMHP mono-(2-carboxymethyl) phthalate; mEHHP mono-(2-ethyl-5-hydroxyhexyl) phthalate; mEOHP mono-2(ethyl-5-oxohexyl) phthalate; mEHP mono-(2-ethylhexyl) phthalate; cr creatinine adjusted, T1 trimester 1, T2 trimester 2, T3 trimester 3.


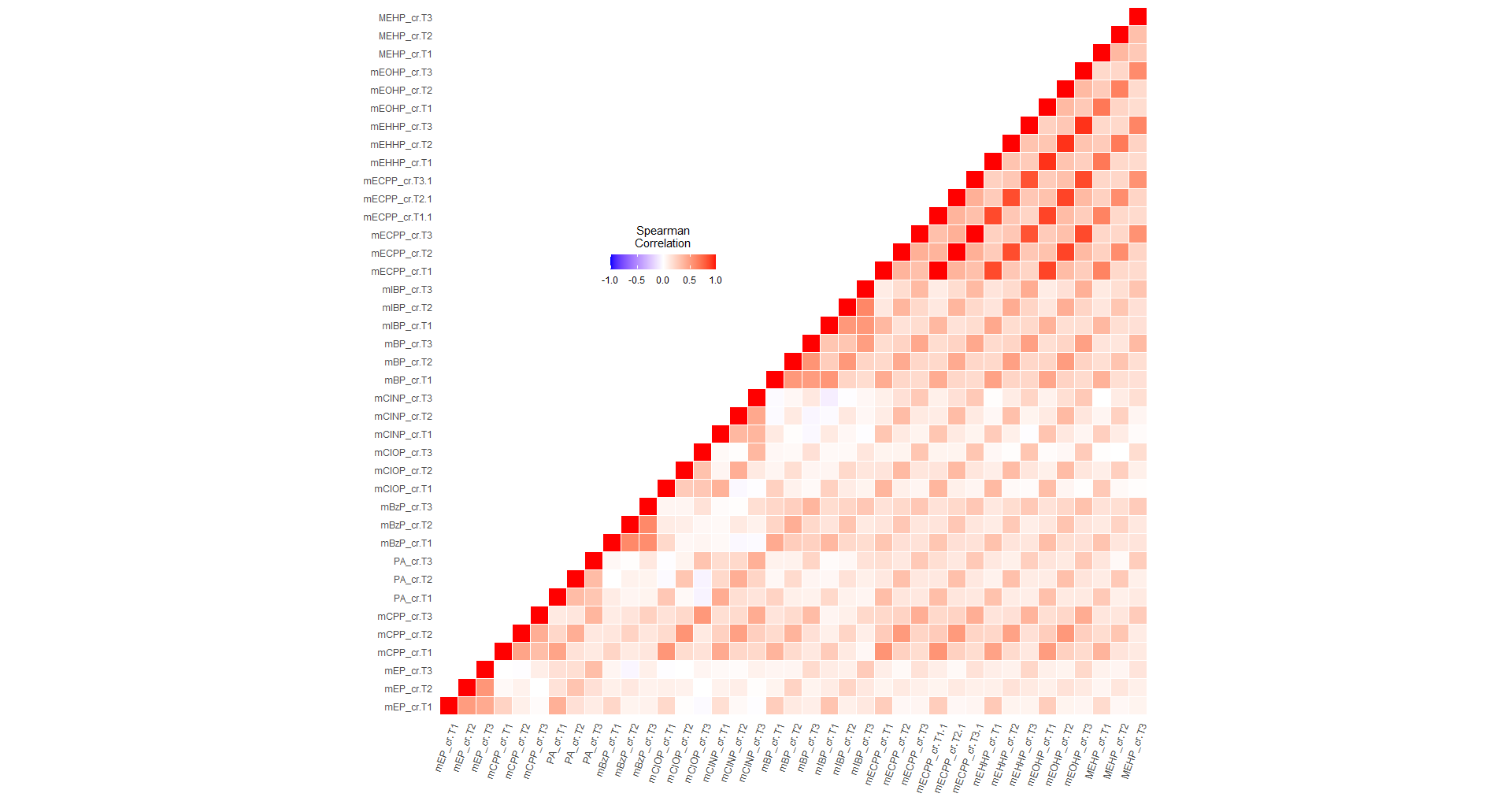


**Text S1.** Details on grouping of the maternal urinary bisphenols and phthalates.

In all trimesters, bisphenol A (BPA) and bisphenol S (BPS) were grouped as total bisphenol. Mono-ethyl phthalate (mEP), mono-n-butyl phthalate (mnBP) and mono-isobutyl phthalate (mIBP) were grouped as low molecular weight phthalates (LMW). High molecular weight phthalate (HMW) was formed of di-(2-ethylhexyl) phthalate (DEHP) and di-*n­-*octyl phthalate (DNOP). DEHP consisted of mono-(2-ethyl-5-carboxypentyl) phthalate (mECPP), mono-(2-carboxymethyl) phthalate (mCMHP), mono-(2-ethyl-5-hydroxyhexyl) phthalate (mEHHP), mono-2(ethyl-5-oxohexyl) phthalate (mEOHP) and mono-(2-ethylhexyl) phthalate (mEHP). DNOP consisted of mono-(3-carboxypropyl) phthalate (mCPP) only.

| **Table S1.** Descriptives and values below the limits of detection per chemical (n = 1091) | | | | | | | | | |
| --- | --- | --- | --- | --- | --- | --- | --- | --- | --- |
|  | **Trimester 1 (n = 1023)** | | | **Trimester 2 (n = 817)** | | | **Trimester 3 (n = 958)** | | |
| **Chemical** | **Concentration (ng/mL)** | **> LOD** | | **Concentration (ng/mL)** | **> LOD** | | **Concentration (ng/mL)** | **> LOD** | |
|  |  | N | % |  | N | % |  | N | % |
| Bisphenol A (BPA) | 0.94 (0.21, 5.52) | 195 | 80.9* | 0.94 (0.18, 6.82) | 185 | 77.4* | 0.87 (0.19, 6.90) | 217 | 77.3* |
| Bisphenol S (BPS) | 0.59 (0.10, 6.49) | 189 | 81.5* | 0.62 (0.11, 6.33) | 166 | 79.7* | 0.60 (0.10, 5.71) | 178 | 81.4* |
| Bisphenol F (BPF ) | 1.00 (0.38, 15.12) | 827 | 19.2 | 0.88 (0.37, 21.32) | 647 | 20.8 | 1.07 (0.37, 13.28) | 757 | 21.0 |
| Bisphenol P (BPP) | 0.25 (0.14, 0.68) | 986 | 0.6 | 0.32 (0.14, 1.02) | 779 | 4.7 | 0.40 (0.14, 1.01) | 919 | 4.1 |
| Bisphenol B (BPB) | 0.66 (0.38, 2.25) | 1010 | 1.3 | 0.73 (0.36, 1.14) | 806 | 1.3 | 0.52 (0.37, 0.63) | 949 | 0.9 |
| Bisphenol Z (BPZ) | 0.44 (0.42, 0.54) | 1020 | 0.3 | 0.54 (0.54, 0.54) | 816 | 0.1 | 0.43 (0.31, 1.11) | 952 | 0.6 |
| Bisphenol AP (BPAP) | 0.55 (0.38, 1.45) | 974 | 4.8 | 0.68 (0.38, 1.47) | 781 | 4.4 | 0.73 (0.37, 2.24) | 887 | 7.4 |
| Bisphenol AF (BPAF) | 0.07 (0.04, 0.28) | 921 | 10.0 | 0.07 (0.04, 0.38) | 705 | 13.7 | 0.11 (0.04, 0.57) | 804 | 16.1 |
| Monomethyl phthalate (mMP) | 4.65 (0.91, 36.46) | 834 | 18.5 | 5.48 (0.49, 55.48) | 713 | 12.7 | 4.71 (0.3, 30.80) | 802 | 16.3 |
| Mono-ethyl phthalate (mEP) | 34.4 (3.28, 755.50) | 2 | 98.8* | 33.34 (2.51, 836.43) | 2 | 99.8* | 33.11 (2.49, 750.91) | 3 | 99.7* |
| Mono-(3-carboxypropyl) phthalate (mCPP) | 0.94 (0.29, 10.08) | 311 | 69.6 | 0.93 (0.25, 8.42) | 157 | 80.8* | 0.96 (0.27, 7.37) | 268 | 72.0* |
| Phthalic acid (PA) | 17.77 (1.19, 142.97) | 78 | 92.4* | 16.4 (1.77, 165.56) | 41 | 95.0* | 17.56 (1.78, 157.9) | 60 | 93.7* |
| Mono-(2-ethyl-5-hydroxyhexyl) phthalate (mEHHP) | 5.92 (0.57, 68.08) | 6 | 99.4* | 5.9 (0.54, 56.28) | 8 | 99.0* | 5.47 (0.44, 53.51) | 5 | 99.5* |
| Mono-benzyl phthalate (mBzP) | 3.81 (0.20, 95.01) | 229 | 77.6* | 3.54 (0.20, 89.41) | 130 | 84.1* | 4.19 (0.20, 93.05) | 218 | 77.2* |
| Mono-cyclohexyl phthalate (mCHP) | 0.13 (0.05, 1.97) | 954 | 6.7 | 0.14 (0.04, 0.59) | 778 | 4.8 | 0.09 (0.05, 1.96) | 907 | 5.3 |
| Mono-octyl phthalate (mOP) | 0.34 (0.05, 1.57) | 973 | 4.9 | 0.94 (0.18, 6.82) | 757 | 7.3 | 0.38 (0.11, 2.91) | 914 | 4.6 |
| Mono-hexyl phthalate (mHxP) | 0.09 (0.02, 1.02) | 722 | 29.4 | 0.62 (0.11, 6.33) | 558 | 31.7 | 0.09 (0.02, 1.80) | 698 | 27.1 |
| Mono-(4-hydroxypentyl phthalate (mHpP) | 0.21 (0.04, 4.05) | 713 | 30.3 | 0.88 (0.37, 21.32) | 498 | 39 | 0.21 (0.05, 3.50) | 658 | 31.3 |
| Mono-(carboxyisooctyl) phthalate (mCIOP) | 1.62 (0.25, 29.60) | 22 | 97.8* | 0.32 (0.14, 1.02) | 13 | 98.4* | 1.64 (0.25, 27.27) | 19 | 98.0* |
| Mono-(carboxyisononyl) phthalate (mCINP) | 1.48 (0.22, 33.58) | 70 | 93.2* | 0.73 (0.36, 1.14) | 64 | 92.2* | 1.35 (0.23, 41.14) | 75 | 92.2* |
| Mono-n-pentyl phthalate (mPeP) | 0.25 (0.21, 0.41) | 1013 | 1.0 | 0.54 (0.54, 0.54) | 806 | 1.3 | 0.42 (0.20, 1.32) | 951 | 0.7 |
| Monoisopropyl phthalate (mIPrP) | 0.23 (0.15, 1.05) | 908 | 11.2 | 0.68 (0.38, 1.47) | 692 | 15.3 | 0.24 (0.15, 1.47) | 842 | 12.1 |
| Mono-n-butyl phthalate (mBP) | 11.0 (0.79, 107.50) | 7 | 99.3* | 0.07 (0.04, 0.38) | 11 | 98.7* | 11.38 (0.93, 124.14) | 11 | 98.9* |
| Mono-isobutyl phthalate (mIBP) | 6.85 (0.57, 64.50) | 15 | 98.5* | 5.48 (0.49, 55.48) | 17 | 97.9* | 7.44 (0.59, 60.82) | 20 | 97.9* |
| Mono-(2-ethyl-5-carboxypentyl) phthalate (mECPP) | 5.9 (0.65, 62.24) | 4 | 99.6* | 33.34 (2.51, 836.43) | 6 | 99.3* | 5.71 (0.62, 54.62) | 5 | 99.5* |
| Mono-(2-carboxymethyl) phthalate (mCMHP) | 2.72 (0.36, 33.78) | 189 | 81.5* | 0.93 (0.25, 8.42) | 83 | 89.8* | 2.60 (0.38, 22.53) | 163 | 83.0* |
| Mono-(7-carboxyheptyl) phthalate (mCHpP) | 1.02 (0.14, 35.71) | 557 | 45.6 | 16.4 (1.77, 165.56) | 422 | 48.3 | 0.86 (0.12, 35.10) | 533 | 44.4 |
| Mono-2(ethyl-5-oxohexyl) phthalate (mEOHP) | 3.44 (0.33, 35.80) | 10 | 99.0* | 5.9 (0.54, 56.28) | 3 | 99.6* | 3.56 (0.32, 33.01) | 6 | 99.4* |
| Mono-isononyl phthalate (mINP) | 1.88 (0.03, 71.08) | 994 | 2.8 | 3.54 (0.20, 89.41) | 786 | 3.8 | 2.42 (0.18, 30.09) | 931 | 2.8 |
| mono-(2-ethylhexyl) phthalate (MEHP) | 2.35 (0.36, 38.35) | 368 | 64.0 | 0.14 (0.04, 0.59) | 282 | 65.5* | 2.48 (0.39, 41.09) | 356 | 62.8* |
| *>50% above limit of detection thus included in analyses. % above LOD is calculated with sample size of the trimester in question. | | | | | | | | | |

| **Table S2.** Non-response analysis of the general characteristics of the study population. | | | |
| --- | --- | --- | --- |
|  | **N = 1091** | **N = 1173** | **P-value** |
| **Maternal characteristics** |  |  |  |
| Maternal age in years, mean (±SD) | 31.8 (5.6) | 31.8 (5.7) | 0.784 |
| Highest education finished, n (%) |  |  | 0.007* |
| High school or less | 375 (34.6) | 337 (30.2) |  |
| College | 117 (10.8) | 140 (12.5) |  |
| Associate degree | 67 (6.2) | 433 (3.8) |  |
| Bachelor’s degree | 245 (22.6) | 263 (23.5) |  |
| Postgraduate degree | 281 (25.9) | 334 (29.9) |  |
| Ethnicity, n (%) |  |  | 0.021* |
| Hispanic | 572 (52.6) | 511 (45.0) |  |
| Non-Hispanic White | 340 (31.3) | 405 (35.7) |  |
| Non-Hispanic Black | 58 (5.3) | 76 (6.7) |  |
| Non-Hispanic Asian | 88 (8.1) | 11 (1.0) |  |
| Other | 10 (0.9) | 26 (2.3) |  |
| Multiple | 19 (1.7) | 511 (45.0) |  |
| Marital status, n (%) |  |  | 0.083 |
| Married/living with a partner | 969 (88.8) | 975 (86.2) |  |
| Divorced/separated | 18 (1.6) | 32 (2.8) |  |
| Single/widowed | 104 (9.5) | 125 (11.0) |  |
| Insured, n (%) |  |  | 0.046* |
| Public | 591 (54.5) | 563 (50.1) |  |
| Private | 494 (45.5) | 560 (49.9) |  |
| Parity, n (%) |  |  | 0.026* |
| Nullipara | 552 (48.8) | 608 (53.7) |  |
| Multipara | 580 (51.2) | 525 (46.3) |  |
| Pre-pregnancy body mass index in kg/m^2^, median (95% range) | 25.0 (18.7, 41.8) | 25.3 (18.8, 40.3) | 0.336 |
| Alcohol use, n (%) |  |  | 0.572 |
| Never | 376 (34.6) | 369 (32.7) |  |
| Stopped in pregnancy | 550 (50.6) | 596 (52.7) |  |
| Continued using alcohol | 160 (14.7) | 165 (14.6) |  |
| Smoking, n (%) |  |  | 0.060 |
| Never | 991 (91.1) | 1027 (90.2) |  |
| Stopped in pregnancy | 79 (7.2) | 103 (9.0) |  |
| Continued in pregnancy | 18 (1.7) | 9 (0.8) |  |
| Hospital of recruitment |  |  | <0.001* |
| Bellevue Hospital, Manhattan | 283 (25.9) | 165 (14.5) |  |
| NYU Brooklyn | 299 (27.4) | 383 (33.6) |  |
| NYU Manhattan | 509 (46.7) | 591 (51.9) |  |
| Values presented as mean (± standard deviation (SD), median (95% range) or number of participants (valid %). Number of missing values per covariate, *n* (%): ethnicity 4 (0.4), education 6 (0.5), pre-pregnancy body mass index 7 (0.6), alcohol use 5 (0.5), smoking 3 (0.3). | | | |

| **Table S3.** Associations of the individual and grouped metabolites with early childhood adiposity outcomes, basic model | | | | | | | | | | | | |
| --- | --- | --- | --- | --- | --- | --- | --- | --- | --- | --- | --- | --- |
|  | **Weight in SDS (n = 1091)** | | | **Body mass index in SDS (n = 672)** | | | **Triceps skinfold in SDS (n = 558)** | | | **Subscapular skinfold in SDS (n = 415)** | | |
| **Chemical** | **Estimate (95% CI)** | **Nominal p-value** | **FDR P-value** | **Estimate (95% CI)** | **Nominal p-value** | **FDR P-value** | **Estimate (95% CI)** | **Nominal p-value** | **FDR P-value** | **Estimate (95% CI)** | **Nominal p-value** | **FDR P-value** |
| BP | 0.04 (-0.02, 0.11) | 0.189 | 0.378 | 0.04 (-0.07, 0.14) | 0.482 | 0.680 | 0.07 (-0.05, 0.18) | 0.244 | 0.562 | -0.08 (-0.21, 0.05) | 0.221 | 0.529 |
| BPA | 0.03 (-0.02, 0.09) | 0.246 | 0.447 | 0.02 (-0.06, 0.11) | 0.600 | 0.749 | 0.05 (-0.04, 0.15) | 0.253 | 0.562 | -0.02 (-0.12, 0.09) | 0.747 | 0.853 |
| BPS | 0.04 (-0.01, 0.09) | 0.103 | 0.229 | 0.06 (-0.03, 0.14) | 0.199 | 0.398 | 0.03 (-0.06, 0.12) | 0.539 | 0.701 | -0.07 (-0.17, 0.03) | 0.180 | 0.529 |
| PA | 0.05 (0.00, 0.10)* | 0.048 | 0.141 | 0.10 (0.02, 0.18)* | 0.014 | 0.071 | -0.07 (-0.15, 0.01) | 0.098 | 0.472 | -0.05 (-0.15, 0.04) | 0.254 | 0.529 |
| LMW | 0.01 (-0.05, 0.06) | 0.780 | 0.918 | -0.06 (-0.15, 0.04) | 0.228 | 0.415 | -0.10 (-0.20, 0.00)* | 0.049 | 0.326 | -0.11 (-0.22, 0.00)* | 0.047 | 0.466 |
| mEP | 0.00 (-0.04, 0.05) | 0.935 | 0.981 | -0.04 (-0.12, 0.03) | 0.279 | 0.465 | -0.06 (-0.15, 0.02) | 0.133 | 0.472 | -0.10 (-0.19, -0.01)* | 0.031 | 0.466 |
| mBP | 0.02 (-0.04, 0.07) | 0.586 | 0.837 | 0.01 (-0.08, 0.10) | 0.845 | 0.889 | -0.14 (-0.24, -0.04)* | 0.006 | 0.125 | -0.02 (-0.14, 0.09) | 0.698 | 0.853 |
| mIBP | 0.02 (-0.04, 0.08) | 0.511 | 0.786 | -0.03 (-0.12, 0.06) | 0.510 | 0.680 | -0.02 (-0.12, 0.07) | 0.659 | 0.701 | 0.00 (-0.10, 0.10) | 0.992 | 0.992 |
| HMW | 0.07 (0.00, 0.13)* | 0.049 | 0.141 | 0.12 (0.01, 0.22)* | 0.033 | 0.112 | 0.04 (-0.07, 0.16) | 0.485 | 0.701 | 0.08 (-0.06, 0.21) | 0.258 | 0.529 |
| DEHP | 0.07 (0.00, 0.13) | 0.041 | 0.141 | 0.12 (0.01, 0.23)* | 0.024 | 0.098 | 0.04 (-0.08, 0.15) | 0.503 | 0.701 | 0.08 (-0.05, 0.21) | 0.245 | 0.529 |
| mECPP | 0.07 (0.00, 0.14)* | 0.037 | 0.141 | 0.14 (0.03, 0.25)* | 0.011 | 0.071 | 0.03 (-0.08, 0.15) | 0.583 | 0.701 | 0.11 (-0.02, 0.24) | 0.100 | 0.529 |
| mCMHP | 0.05 (0.01, 0.10)* | 0.012 | 0.141 | 0.10 (0.04, 0.17)* | 0.006 | 0.058 | 0.02 (-0.06, 0.10) | 0.598 | 0.701 | 0.05 (-0.04, 0.13) | 0.265 | 0.529 |
| mEHHP | 0.07 (0.01, 0.14)* | 0.027 | 0.141 | 0.09 (-0.02, 0.16) | 0.108 | 0.269 | 0.03 (-0.08, 0.14) | 0.599 | 0.701 | 0.07 (-0.06, 0.19) | 0.309 | 0.562 |
| mEOHP | 0.07 (0.00, 0.13)* | 0.043 | 0.141 | 0.10 (0.00, 0.17) | 0.055 | 0.157 | 0.05 (-0.06, 0.14) | 0.346 | 0.692 | 0.06 (-0.07, 0.18) | 0.369 | 0.587 |
| MEHP | 0.04 (-0.01, 0.08) | 0.102 | 0.229 | 0.10 (0.03, 0.22)* | 0.006 | 0.058 | 0.02 (-0.06, 0.10) | 0.610 | 0.701 | 0.06 (-0.03, 0.15) | 0.185 | 0.529 |
| DNOP | 0.01 (-0.04, 0.06) | 0.733 | 0.916 | 0.01 (-0.07, 0.10) | 0.758 | 0.842 | 0.02 (-0.07, 0.11) | 0.701 | 0.701 | -0.02 (-0.12, 0.10) | 0.767 | 0.853 |
| mCPP | 0.01 (-0.04, 0.06) | 0.733 | 0.916 | 0.01 (-0.07, 0.10) | 0.758 | 0.842 | 0.02 (-0.07, 0.11) | 0.701 | 0.701 | -0.02 (-0.12, 0.10) | 0.767 | 0.853 |
| mBzP | 0.00 (-0.03, 0.03) | 0.981 | 0.981 | 0.03 (-0.01, 0.08) | 0.176 | 0.391 | -0.06 (-0.11, -0.01)* | 0.014 | 0.136 | 0.00 (-0.06, 0.06) | 0.988 | 0.992 |
| mCIOP | 0.02 (-0.03, 0.07) | 0.485 | 0.786 | 0.00 (-0.09, 0.09) | 0.991 | 0.991 | 0.07 (-0.03, 0.16) | 0.165 | 0.472 | 0.05 (-0.06, 0.15) | 0.391 | 0.587 |
| mCINP | 0.00 (-0.05, 0.05) | 0.936 | 0.981 | 0.04 (-0.04, 0.11) | 0.365 | 0.562 | 0.06 (-0.02, 0.15) | 0.165 | 0.472 | -0.04 (-0.14, 0.06) | 0.411 | 0.587 |
| Values are obtained from linear mixed-effect models and represent the SDS change in overall adiposity outcomes (95% confidence interval (CI)) between birth and 4 years per natural log increase in the chemical exposure. Outcome SDs are sex-specific z-scores for age according to World Health Organization growth charts. BP total bipshenol; BPA bisphenol A; BPS bisphenol S; PA phthalic acid; LMW low molecular weight phthalate; mEP mono-ethyl phthalate; mnBP mono-n-butyl phthalate; mIBP mono-isobutyl phthalate; HMW high molecular weight phthalate; DEHP di-(2-ethylhexyl) phthalate; mECPP mono-(2-ethyl-5-carboxypentyl) phthalate; mCMHP mono-(2-carboxymethyl) phthalate; mEHHP mono-(2-ethyl-5-hydroxyhexyl) phthalate; mEOHP mono-2(ethyl-5-oxohexyl) phthalate; mEHP mono-(2-ethylhexyl) phthalate; DNOP di-*n*-octyl phthalate; mCPP mono-(3-carboxypropyl) phthalate; mBzP mono-benzyl phthalate; mCIOP mono-(carboxyisooctyl) phthalate; mCINP mono-(carboxyisononyl) phthalate. FDR False Discovery Rate. *Nominal significant p-value <0.05 | | | | | | | | | | | | |

| **Table S4.** Associations of the individual and grouped trimester-specific metabolites with early childhood weight between birth and 4 years, adjusted model (n = 1091) | | | | | | | | | |
| --- | --- | --- | --- | --- | --- | --- | --- | --- | --- |
|  | **Trimester 1** | | | **Trimester 2** | | | **Trimester 3** | | |
| **Chemical** | **Estimate (95% CI)** | **Nominal p-value** | **FDR P-value** | **Estimate (95% CI)** | **Nominal p-value** | **FDR P-value** | **Estimate (95% CI)** | **Nominal p-value** | **FDR P-value** |
| BP | 0.01 (-0.04, 0.06) | 0.655 | 0.929 | 0.10 (0.04, 0.16)** | 0.002 | 0.031 | 0.07 (0.01, 0.12)** | 0.015 | 0.151 |
| BPA | 0.03 (-0.01, 0.08) | 0.157 | 0.929 | 0.07 (0.02, 0.12)** | 0.004 | 0.043 | 0.06 (0.02, 0.11)** | 0.007 | 0.141 |
| BPS | -0.02 (-0.06, 0.02) | 0.295 | 0.929 | 0.05 (0.00, 0.09) | 0.060 | 0.307 | 0.04 (-0.01, 0.08) | 0.094 | 0.525 |
| PA | 0.01 (-0.03, 0.04) | 0.700 | 0.929 | 0.01 (-0.04, 0.05) | 0.774 | 0.826 | -0.03 (-0.01, 0.07) | 0.134 | 0.536 |
| LMW | 0.00 (-0.05, 0.05) | 0.915 | 0.929 | 0.04 (-0.02 ,0.10) | 0.215 | 0.376 | -0.01 (-0.07, 0.04) | 0.584 | 0.687 |
| mEP | 0.00 (-0.05, 0.04) | 0.847 | 0.929 | 0.03 (-0.02, 0.08) | 0.282 | 0.376 | -0.01 (-0.06, 0.03) | 0.516 | 0.645 |
| mBP | 0.00 (-0.05, 0.05) | 0.860 | 0.929 | 0.03 (-0.03, 0.09) | 0.301 | 0.376 | -0.04 (-0.09, 0.02) | 0.177 | 0.588 |
| mIBP | -0.01 (-0.06, 0.03) | 0.636 | 0.929 | 0.02 (-0.03, 0.07) | 0.357 | 0.421 | 0.00 (-0.04, 0.05) | 0.916 | 0.916 |
| HMW | 0.01 (-0.05, 0.07) | 0.696 | 0.929 | 0.05 (-0.02, 0.11) | 0.171 | 0.376 | 0.03 (-0.03, 0.10) | 0.342 | 0.617 |
| DEHP | 0.01 (-0.04, 0.07) | 0.651 | 0.929 | 0.05 (-0.02, 0.11) | 0.163 | 0.376 | 0.03 (-0.03, 0.09) | 0.370 | 0.617 |
| mECPP | 0.03 (-0.03, 0.08) | 0.311 | 0.929 | 0.04 (-0.02, 0.10) | 0.193 | 0.376 | 0.03 (-0.03, 0.09) | 0.320 | 0.617 |
| mCMHP | 0.02 (-0.02, 0.05) | 0.415 | 0.929 | 0.05 (0.00, 0.09) | 0.064 | 0.307 | 0.02 (-0.02, 0.06) | 0.228 | 0.617 |
| mEHHP | 0.02 (-0.04, 0.07) | 0.502 | 0.929 | 0.05 (-0.01, 0.11) | 0.090 | 0.307 | 0.02 (-0.04, 0.08) | 0.482 | 0.642 |
| mEOHP | 0.02 (-0.03, 0.07) | 0.535 | 0.929 | 0.06 (-0.01, 0.12) | 0.092 | 0.307 | 0.03 (-0.03, 0.09) | 0.278 | 0.617 |
| MEHP | 0.00 (-0.04, 0.04) | 0.909 | 0.929 | 0.02 (-0.02, 0.07) | 0.296 | 0.376 | 0.02 (-0.02, 0.06) | 0.408 | 0.627 |
| DNOP | -0.01 (-0.06, 0.04) | 0.813 | 0.929 | 0.03 (-0.02, 0.10) | 0.292 | 0.376 | 0.01 (-0.04, 0.06) | 0.729 | 0.768 |
| mCPP | -0.01 (-0.06, 0.04) | 0.813 | 0.929 | 0.03 (-0.03, 0.10) | 0.292 | 0.376 | 0.01 (-0.04, 0.06) | 0.729 | 0.768 |
| mBzP | 0.00 (-0.02, 0.03) | 0.929 | 0.929 | 0.00 (-0.03, 0.03) | 0.978 | 0.978 | -0.01 (-0.04, 0.01) | 0.314 | 0.617 |
| mCIOP | -0.02 (-0.07, 0.03) | 0.428 | 0.929 | 0.01 (-0.05, 0.06) | 0.785 | 0.826 | 0.02 (-0.03, 0.07) | 0.477 | 0.642 |
| mCINP | 0.01 (-0.04, 0.05) | 0.707 | 0.929 | 0.03 (-0.02, 0.08) | 0.280 | 0.376 | 0.04 (-0.01, 0.08) | 0.105 | 0.525 |
| Values are obtained from linear mixed-effect models and represent the SDS change in weight (95% confidence interval (CI)) between birth and 4 years per natural log increase in the chemical exposure. Outcome SDs are sex-specific z-scores for age according to World Health Organization growth charts. Model is adjusted for race, education, marital status, insurance, parity, body mass index, alcohol and tabacco use. BP total bipshenol; BPA bisphenol A; BPS bisphenol S; PA phthalic acid; LMW low molecular weight phthalate; mEP mono-ethyl phthalate; mnBP mono-n-butyl phthalate; mIBP mono-isobutyl phthalate; HMW high molecular weight phthalate; DEHP di-(2-ethylhexyl) phthalate; mECPP mono-(2-ethyl-5-carboxypentyl) phthalate; mCMHP mono-(2-carboxymethyl) phthalate; mEHHP mono-(2-ethyl-5-hydroxyhexyl) phthalate; mEOHP mono-2(ethyl-5-oxohexyl) phthalate; mEHP mono-(2-ethylhexyl) phthalate; DNOP di-*n*-octyl phthalate; mCPP mono-(3-carboxypropyl) phthalate; mBzP mono-benzyl phthalate; mCIOP mono-(carboxyisooctyl) phthalate; mCINP mono-(carboxyisononyl) phthalate. **False Discovery Rate (FDR) adjusted significant p-value <0.05 | | | | | | | | | |

| **Table S5.** Associations of the individual and grouped trimester-specific metabolites with early childhood body mass index between 1 and 4 years, adjusted model (n = 672) | | | | | | | | | |
| --- | --- | --- | --- | --- | --- | --- | --- | --- | --- |
|  | **Trimester 1** | | | **Trimester 2** | | | **Trimester 3** | | |
| **Chemical** | **Estimate (95% CI)** | **Nominal p-value** | **FDR P-value** | **Estimate (95% CI)** | **Nominal p-value** | **FDR P-value** | **Estimate (95% CI)** | **Nominal p-value** | **FDR P-value** |
| BP | -0.03 (-0.12, 0.05) | 0.456 | 0.772 | 0.07 (-0.03, 0.18) | 0.153 | 0.313 | 0.09 (0.01, 0.18)* | 0.037 | 0.146 |
| BPA | -0.02 (-0.09, 0.06) | 0.625 | 0.859 | 0.06 (-0.01, 0.14) | 0.113 | 0.313 | 0.10 (0.02, 0.17)* | 0.009 | 0.146 |
| BPS | -0.05 (-0.12, 0.02) | 0.167 | 0.685 | 0.00 (-0.07, 0.08) | 0.909 | 0.909 | 0.04 (-0.02, 0.11) | 0.199 | 0.408 |
| PA | 0.03 (-0.03, 0.09) | 0.322 | 0.685 | 0.03 (-0.05, 0.11) | 0.504 | 0.792 | 0.07 (0.01, 0.13)* | 0.034 | 0.146 |
| LMW | -0.08 (-0.16, 0.00) | 0.062 | 0.685 | -0.02 (-0.12, 0.07) | 0.647 | 0.792 | -0.04 (-0.13, 0.04) | 0.297 | 0.408 |
| mEP | -0.06 (-0.12, 0.01) | 0.108 | 0.685 | -0.01 (-0.09, 0.07) | 0.881 | 0.909 | -0.03 (-0.10, 0.03) | 0.337 | 0.421 |
| mBP | -0.04 (-0.12, 0.04) | 0.342 | 0.685 | -0.02 (-0.11, 0.07) | 0.673 | 0.792 | -0.05 (-0.13, 0.04) | 0.274 | 0.408 |
| mIBP | -0.07 (-0.14, 0.01) | 0.072 | 0.685 | 0.00 (-0.08, 0.07) | 0.906 | 0.909 | -0.04 (-0.11, 0.03) | 0.306 | 0.408 |
| HMW | 0.00 (-0.09, 0.09) | 0.997 | 0.997 | 0.09 (-0.02, 0.20) | 0.112 | 0.313 | 0.08 (-0.03, 0.18) | 0.145 | 0.408 |
| DEHP | 0.00 (-0.09, 0.09) | 0.929 | 0.974 | 0.09 (-0.01, 0.20) | 0.094 | 0.313 | 0.07 (-0.03, 0.18) | 0.163 | 0.408 |
| mECPP | 0.03 (-0.06, 0.12) | 0.500 | 0.786 | 0.08 (-0.03, 0.18) | 0.147 | 0.313 | 0.06 (-0.04, 0.15) | 0.271 | 0.408 |
| mCMHP | 0.04 (-0.02, 0.10) | 0.193 | 0.685 | 0.08 (0.00, 0.16) | 0.062 | 0.313 | 0.07 (0.01, 0.14)* | 0.023 | 0.146 |
| mEHHP | -0.01 (-0.10, 0.07) | 0.767 | 0.950 | 0.07 (-0.03, 0.17) | 0.157 | 0.313 | 0.03 (-0.06, 0.13) | 0.508 | 0.565 |
| mEOHP | 0.01 (-0.07, 0.09) | 0.892 | 0.974 | 0.09 (-0.01, 0.19) | 0.081 | 0.313 | 0.04 (-0.05, 0.13) | 0.389 | 0.458 |
| MEHP | 0.01 (-0.05, 0.07) | 0.777 | 0.950 | 0.10 (0.02, 0.18) | 0.012 | 0.231 | 0.06 (0.00, 0.13) | 0.065 | 0.218 |
| DNOP | -0.04 (-0.12, 0.04) | 0.318 | 0.685 | 0.03 (-0.08, 0.14) | 0.621 | 0.792 | 0.05 (-0.03, 0.14) | 0.227 | 0.408 |
| mCPP | -0.04 (-0.12, 0.04) | 0.318 | 0.685 | 0.03 (-0.08, 0.14) | 0.621 | 0.792 | 0.05 (-0.03, 0.14) | 0.227 | 0.408 |
| mBzP | 0.00 (-0.04, 0.04) | 0.832 | 0.964 | 0.04 (-0.02, 0.09) | 0.185 | 0.337 | 0.01 (-0.03, 0.05) | 0.686 | 0.686 |
| mCIOP | -0.05 (-0.12, 0.03) | 0.243 | 0.685 | -0.03 (-0.12, 0.06) | 0.552 | 0.792 | 0.02 (-0.06, 0.10) | 0.672 | 0.686 |
| mCINP | 0.04 (-0.03, 0.11) | 0.250 | 0.685 | 0.07 (-0.01, 0.15) | 0.098 | 0.313 | 0.08 (0.01, 0.15)* | 0.029 | 0.146 |
| Values are obtained from linear mixed-effect models and represent the SDS change in body mass index (95% confidence interval (CI)) between 1 and 4 years per natural log increase in the chemical exposure. Outcome SDs are sex-specific z-scores for age according to World Health Organization growth charts. Model is adjusted for race, education, marital status, insurance, parity, body mass index, alcohol and tabacco use. BP total bipshenol; BPA bisphenol A; BPS bisphenol S; PA phthalic acid; LMW low molecular weight phthalate; mEP mono-ethyl phthalate; mnBP mono-n-butyl phthalate; mIBP mono-isobutyl phthalate; HMW high molecular weight phthalate; DEHP di-(2-ethylhexyl) phthalate; mECPP mono-(2-ethyl-5-carboxypentyl) phthalate; mCMHP mono-(2-carboxymethyl) phthalate; mEHHP mono-(2-ethyl-5-hydroxyhexyl) phthalate; mEOHP mono-2(ethyl-5-oxohexyl) phthalate; mEHP mono-(2-ethylhexyl) phthalate; DNOP di-*n*-octyl phthalate; mCPP mono-(3-carboxypropyl) phthalate; mBzP mono-benzyl phthalate; mCIOP mono-(carboxyisooctyl) phthalate; mCINP mono-(carboxyisononyl) phthalate. FDR False Discovery Rate. | | | | | | | | | |

| **Table S6.** Associations of the individual and grouped trimester-specific metabolites with early childhood triceps skinfold between 1 and 4 years, adjusted model (n = 558) | | | | | | | | | |
| --- | --- | --- | --- | --- | --- | --- | --- | --- | --- |
|  | **Trimester 1** | | | **Trimester 2** | | | **Trimester 3** | | |
| **Chemical** | **Estimate (95% CI)** | **Nominal p-value** | **FDR P-value** | **Estimate (95% CI)** | **Nominal p-value** | **FDR P-value** | **Estimate (95% CI)** | **Nominal p-value** | **FDR P-value** |
| BP | 0.04 (-0.05, 0.13) | 0.413 | 0.644 | -0.02 (-0.13, 0.09) | 0.707 | 0.884 | 0.04 (-0.06, 0.13) | 0.471 | 0.902 |
| BPA | 0.00 (-0.08, 0.08) | 0.966 | 0.966 | -0.02 (-0.11, 0.06) | 0.562 | 0.864 | 0.07 (-0.01, 0.15) | 0.111 | 0.557 |
| BPS | 0.05 (-0.03, 0.12) | 0.216 | 0.540 | -0.01 (-0.09, 0.08) | 0.894 | 0.985 | 0.01 (-0.06, 0.09) | 0.780 | 0.902 |
| PA | -0.03 (-0.09, 0.04) | 0.436 | 0.644 | 0.00 (-0.09, 0.09) | 0.985 | 0.985 | -0.07 (-0.13, 0.00)* | 0.045 | 0.306 |
| LMW | 0.00 (-0.09, 0.08) | 0.927 | 0.966 | -0.02 (-0.13, 0.08) | 0.685 | 0.884 | -0.07 (-0.16, 0.03) | 0.163 | 0.652 |
| mEP | -0.01 (-0.08, 0.06) | 0.841 | 0.942 | -0.03 (-0.12, 0.06) | 0.492 | 0.820 | -0.07 (-0.14, 0.00)* | 0.046 | 0.306 |
| mBP | 0.01 (-0.08, 0.10) | 0.848 | 0.942 | -0.01 (-0.10, 0.08) | 0.855 | 0.985 | -0.09 (-0.18, 0.00)* | 0.041 | 0.306 |
| mIBP | 0.02 (-0.06, 0.09) | 0.680 | 0.850 | 0.02 (-0.06, 0.10) | 0.609 | 0.870 | 0.02 (-0.06, 0.09) | 0.650 | 0.902 |
| HMW | 0.10 (0.00, 0.20) | 0.052 | 0.258 | 0.09 (-0.02, 0.20) | 0.126 | 0.442 | 0.01 (-0.10, 0.13) | 0.857 | 0.902 |
| DEHP | 0.10 (0.00, 0.20) | 0.057 | 0.258 | 0.09 (-0.02, 0.20) | 0.112 | 0.442 | 0.01 (-0.11, 0.12) | 0.928 | 0.928 |
| mECPP | 0.11 (0.01, 0.20)* | 0.030 | 0.258 | 0.08 (-0.02, 0.19) | 0.133 | 0.442 | 0.01 (-0.10, 0.12) | 0.855 | 0.902 |
| mCMHP | 0.02 (-0.04, 0.09) | 0.507 | 0.676 | 0.09 (0.00, 0.18) | 0.045 | 0.442 | 0.03 (-0.04, 0.10) | 0.407 | 0.902 |
| mEHHP | 0.09 (0.00, 0.18) | 0.056 | 0.258 | 0.06 (-0.04, 0.16) | 0.260 | 0.566 | -0.02 (-0.12, 0.08) | 0.734 | 0.902 |
| mEOHP | 0.08 (-0.01, 0.17) | 0.077 | 0.258 | 0.10 (-0.01, 0.21) | 0.084 | 0.442 | -0.03 (-0.13, 0.07) | 0.539 | 0.902 |
| MEHP | 0.04 (-0.02, 0.11) | 0.208 | 0.540 | 0.07 (-0.01, 0.15) | 0.080 | 0.442 | 0.01 (-0.06, 0.08) | 0.822 | 0.902 |
| DNOP | 0.04 (-0.06, 0.13) | 0.451 | 0.644 | 0.06 (-0.05, 0.17) | 0.312 | 0.566 | 0.04 (-0.06, 0.13) | 0.427 | 0.902 |
| mCPP | 0.04 (-0.06, 0.13) | 0.451 | 0.644 | 0.06 (-0.05, 0.17) | 0.312 | 0.566 | 0.04 (-0.06, 0.13) | 0.427 | 0.902 |
| mBzP | -0.02 (-0.07, 0.02) | 0.302 | 0.644 | 0.03 (-0.02, 0.09) | 0.258 | 0.566 | -0.01 (-0.06, 0.03) | 0.557 | 0.902 |
| mCIOP | 0.08 (0.00, 0.16) | 0.066 | 0.258 | 0.00 (-0.09, 0.10) | 0.948 | 0.985 | 0.01 (-0.08, 0.09) | 0.850 | 0.902 |
| mCINP | 0.04 (-0.04, 0.12) | 0.374 | 0.644 | 0.07 (-0.03, 0.16) | 0.165 | 0.471 | 0.03 (-0.05, 0.11) | 0.507 | 0.902 |
| Values are obtained from linear mixed-effect models and represent the SDS change in triceps skinfold thickness (95% confidence interval (CI)) between 1 and 4 years per natural log increase in the chemical exposure. Outcome SDs are sex-specific z-scores for age according to World Health Organization growth charts. Model is adjusted for race, education, marital status, insurance, parity, body mass index, alcohol and tabacco use. BP total bipshenol; BPA bisphenol A; BPS bisphenol S; PA phthalic acid; LMW low molecular weight phthalate; mEP mono-ethyl phthalate; mnBP mono-n-butyl phthalate; mIBP mono-isobutyl phthalate; HMW high molecular weight phthalate; DEHP di-(2-ethylhexyl) phthalate; mECPP mono-(2-ethyl-5-carboxypentyl) phthalate; mCMHP mono-(2-carboxymethyl) phthalate; mEHHP mono-(2-ethyl-5-hydroxyhexyl) phthalate; mEOHP mono-2(ethyl-5-oxohexyl) phthalate; mEHP mono-(2-ethylhexyl) phthalate; DNOP di-*n*-octyl phthalate; mCPP mono-(3-carboxypropyl) phthalate; mBzP mono-benzyl phthalate; mCIOP mono-(carboxyisooctyl) phthalate; mCINP mono-(carboxyisononyl) phthalate. FDR False Discovery Rate *Nominal significant p-value <0.05 | | | | | | | | | |

| **Table S7.** Associations of the individual and grouped trimester-specific metabolites with early childhood subscapular skinfold between 1 and 4 years, adjusted model (n = 415) | | | | | | | | | |
| --- | --- | --- | --- | --- | --- | --- | --- | --- | --- |
|  | **Trimester 1** | | | **Trimester 2** | | | **Trimester 3** | | |
| **Chemical** | **Estimate (95% CI)** | **Nominal p-value** | **FDR P-value** | **Estimate (95% CI)** | **Nominal p-value** | **FDR P-value** | **Estimate (95% CI)** | **Nominal p-value** | **FDR P-value** |
| BP | 0.00 (-0.10, 0.10) | 0.991 | 0.991 | -0.05 (-0.18, 0.08) | 0.436 | 0.791 | -0.11 (-0.22, 0.00) | 0.063 | 0.316 |
| BPA | 0.03 (-0.05, 0.12) | 0.487 | 0.762 | -0.04 (-0.14, 0.06) | 0.427 | 0.791 | -0.01 (-0.09, 0.08) | 0.894 | 0.938 |
| BPS | -0.04 (-0.12, 0.04) | 0.289 | 0.577 | -0.05 (-0.15, 0.05) | 0.341 | 0.791 | -0.09 (-0.18, 0.00)* | 0.042 | 0.280 |
| PA | -0.05 (-0.12, 0.01) | 0.118 | 0.577 | 0.00 (-0.09, 0.10) | 0.932 | 0.932 | -0.06 (-0.14, 0.01) | 0.106 | 0.426 |
| LMW | -0.03 (-0.12, 0.06) | 0.495 | 0.762 | -0.05 (-0.16, 0.07) | 0.429 | 0.791 | -0.12 (-0.22, -0.02)* | 0.027 | 0.266 |
| mEP | -0.03 (-0.11, 0.05) | 0.430 | 0.762 | -0.06 (-0.16, 0.04) | 0.215 | 0.791 | -0.12 (-0.20, -0.04)* | 0.003 | 0.060 |
| mBP | 0.02 (-0.07, 0.11) | 0.670 | 0.933 | 0.02 (-0.09, 0.12) | 0.758 | 0.930 | -0.06 (-0.17, 0.05) | 0.295 | 0.555 |
| mIBP | 0.01 (-0.07, 0.09) | 0.811 | 0.954 | 0.05 (-0.04, 0.14) | 0.287 | 0.791 | 0.00 (-0.08, 0.08) | 0.938 | 0.938 |
| HMW | 0.06 (-0.05, 0.17) | 0.260 | 0.577 | 0.04 (-0.09, 0.16) | 0.579 | 0.791 | -0.07 (-0.20, 0.07) | 0.333 | 0.555 |
| DEHP | 0.06 (-0.04, 0.17) | 0.249 | 0.577 | 0.04 (-0.09, 0.16) | 0.554 | 0.791 | -0.07 (-0.20, 0.06) | 0.312 | 0.555 |
| mECPP | 0.11 (0.00, 0.21)* | 0.049 | 0.577 | 0.06 (-0.06, 0.19) | 0.344 | 0.791 | -0.07 (-0.20, 0.06) | 0.289 | 0.555 |
| mCMHP | 0.01 (-0.06, 0.08) | 0.747 | 0.933 | 0.06 (-0.04, 0.15) | 0.243 | 0.791 | 0.02 (-0.06, 0.09) | 0.709 | 0.938 |
| mEHHP | 0.08 (-0.02, 0.17) | 0.116 | 0.577 | 0.03 (-0.08, 0.14) | 0.593 | 0.791 | -0.09 (-0.20, 0.03) | 0.141 | 0.469 |
| mEOHP | 0.06 (-0.03, 0.16) | 0.220 | 0.577 | 0.05 (-0.07, 0.17) | 0.413 | 0.791 | -0.07 (-0.18, 0.05) | 0.262 | 0.555 |
| MEHP | 0.04 (-0.03, 0.12) | 0.256 | 0.577 | 0.04 (-0.06, 0.13) | 0.444 | 0.791 | -0.03 (-0.12, 0.05) | 0.430 | 0.649 |
| DNOP | 0.00 (-0.10, 0.10) | 0.964 | 0.991 | 0.01 (-0.11, 0.14) | 0.837 | 0.930 | -0.01 (-0.12, 0.10) | 0.816 | 0.938 |
| mCPP | 0.00 (-0.10, 0.10) | 0.964 | 0.991 | 0.01 (-0.11, 0.14) | 0.837 | 0.930 | -0.01 (-0.12, 0.10) | 0.816 | 0.938 |
| mBzP | -0.01 (-0.06, 0.04) | 0.729 | 0.933 | 0.05 (-0.01, 0.11) | 0.093 | 0.791 | -0.02 (-0.07, 0.03) | 0.454 | 0.649 |
| mCIOP | 0.06 (-0.03, 0.15) | 0.235 | 0.577 | -0.03 (-0.15, 0.08) | 0.579 | 0.791 | 0.05 (-0.05, 0.15) | 0.314 | 0.555 |
| mCINP | -0.07 (-0.16, 0.01) | 0.105 | 0.577 | 0.01 (-0.10, 0.11) | 0.925 | 0.932 | 0.01 (-0.08, 0.10) | 0.870 | 0.938 |
| Values are obtained from linear mixed-effect models and represent the SDS change in subscapular skinfold thickness (95% confidence interval (CI)) between 1 and 4 years per natural log increase in the chemical exposure. Model is adjusted for race, education, marital status, insurance, parity, body mass index, alcohol and tabacco use. BP total bipshenol; BPA bisphenol A; BPS bisphenol S; PA phthalic acid; LMW low molecular weight phthalate; mEP mono-ethyl phthalate; mnBP mono-n-butyl phthalate; mIBP mono-isobutyl phthalate; HMW high molecular weight phthalate; DEHP di-(2-ethylhexyl) phthalate; mECPP mono-(2-ethyl-5-carboxypentyl) phthalate; mCMHP mono-(2-carboxymethyl) phthalate; mEHHP mono-(2-ethyl-5-hydroxyhexyl) phthalate; mEOHP mono-2(ethyl-5-oxohexyl) phthalate; mEHP mono-(2-ethylhexyl) phthalate; DNOP di-*n*-octyl phthalate; mCPP mono-(3-carboxypropyl) phthalate; mBzP mono-benzyl phthalate; mCIOP mono-(carboxyisooctyl) phthalate; mCINP mono-(carboxyisononyl) phthalate. FDR False Discovery Rate. *Nominal significant p-value <0.05 | | | | | | | | | |

| **Table S8.** Effect of exposures on early childhood weight at each time point, adjusted model (n = 1091) | | | | | | | | | | | | |
| --- | --- | --- | --- | --- | --- | --- | --- | --- | --- | --- | --- | --- |
|  | **1 year** | | | **2 year** | | | **3 year** | | | **4 year** | | |
| **Chemical** | **Estimate (95% CI)** | **Nominal p-value** | **FDR P-value** | **Estimate (95% CI)** | **Nominal p-value** | **FDR P-value** | **Estimate (95% CI)** | **Nominal p-value** | **FDR P-value** | **Estimate (95% CI)** | **Nominal p-value** | **FDR P-value** |
| BP | 0.02 (-0.06, 0.12) | 0.598 | 0.854 | -0.01 (-0.11, 0.10) | 0.890 | 0.976 | 0.02 (-0.10, 0.15) | 0.736 | 0.774 | 0.05 (-0.07, 0.17) | 0.446 | 0.696 |
| BPA | 0.00 (-0.07, 0.08) | 0.903 | 0.913 | -0.05 (-0.14, 0.04) | 0.262 | 0.798 | 0.03 (-0.07, 0.13) | 0.548 | 0.774 | 0.07 (-0.03, 0.18) | 0.157 | 0.349 |
| BPS | 0.02 (-0.06, 0.09) | 0.582 | 0.854 | 0.04 (-0.05, 0.12) | 0.391 | 0.798 | 0.02 (-0.09, 0.12) | 0.725 | 0.774 | 0.00 (-0.09, 0.10) | 0.931 | 0.962 |
| PA | 0.05 (-0.02, 0.12) | 0.149 | 0.803 | 0.11 (0.03, 0.19) | 0.006* | 0.062 | 0.11 (0.01, 0.21) | 0.030* | 0.297 | 0.09 (-0.01, 0.19) | 0.087 | 0.218 |
| LMW | -0.03 (-0.11, 0.05) | 0.485 | 0.854 | -0.03 (-0.12, 0.06) | 0.507 | 0.798 | -0.03 (-0.13, 0.08) | 0.628 | 0.774 | -0.01 (-0.12, 0.09) | 0.817 | 0.962 |
| mEP | 0.00 (-0.07, 0.07) | 0.913 | 0.913 | -0.02 (-0.09, 0.06) | 0.675 | 0.798 | 0.00 (-0.08, 0.09) | 0.921 | 0.921 | 0.00 (-0.09, 0.08) | 0.937 | 0.962 |
| mBP | -0.07 (-0.14, 0.02) | 0.118 | 0.803 | -0.02 (-0.11, 0.07) | 0.678 | 0.798 | -0.09 (-0.21, 0.02) | 0.097 | 0.312 | -0.02 (-0.12, 0.09) | 0.761 | 0.962 |
| mIBP | -0.05 (-0.13, 0.03) | 0.226 | 0.803 | -0.02 (-0.11, 0.06) | 0.588 | 0.798 | -0.02 (-0.14, 0.10) | 0.725 | 0.774 | -0.01 (-0.12, 0.11) | 0.924 | 0.962 |
| HMW | -0.03 (-0.10, 0.06) | 0.507 | 0.854 | 0.02 (-0.08, 0.13) | 0.649 | 0.798 | 0.10 (-0.02, 0.22) | 0.109 | 0.312 | 0.16 (0.04, 0.28) | 0.011* | 0.047* |
| DEHP | -0.03 (-0.11, 0.06) | 0.496 | 0.854 | 0.02 (-0.08, 0.13) | 0.666 | 0.798 | 0.10 (-0.02, 0.22) | 0.093 | 0.312 | 0.15 (0.03, 0.27) | 0.012* | 0.047* |
| mECPP | -0.01 (-0.09, 0.08) | 0.794 | 0.913 | 0.05 (-0.06, 0.16) | 0.341 | 0.798 | 0.12 (0.00, 0.25) | 0.048* | 0.312 | 0.19 (0.07, 0.31) | 0.003* | 0.028* |
| mCMHP | 0.04 (-0.02, 0.10) | 0.175 | 0.803 | 0.07 (0.00, 0.14) | 0.045* | 0.299 | 0.14 (0.06, 0.22) | 0.001* | 0.018* | 0.16 (0.07, 0.24) | 0.001* | 0.003* |
| mEHHP | -0.07 (-0.14, 0.02) | 0.136 | 0.803 | 0.00 (-0.10, 0.10) | 0.976 | 0.976 | 0.08 (-0.03, 0.20) | 0.167 | 0.370 | 0.11 (-0.01, 0.22) | 0.077 | 0.218 |
| mEOHP | -0.06 (-0.12, 0.04) | 0.241 | 0.803 | 0.00 (-0.10, 0.11) | 0.931 | 0.976 | 0.09 (-0.03, 0.20) | 0.153 | 0.370 | 0.14 (0.02, 0.25) | 0.022* | 0.075 |
| MEHP | 0.01 (-0.07, 0.07) | 0.850 | 0.913 | 0.03 (-0.04, 0.10) | 0.403 | 0.798 | 0.07 (-0.01, 0.15) | 0.088 | 0.312 | 0.11 (0.03, 0.19) | 0.006* | 0.039* |
| DNOP | 0.02 (-0.06, 0.10) | 0.588 | 0.854 | 0.05 (-0.03, 0.14) | 0.242 | 0.798 | 0.05 (-0.05, 0.16) | 0.324 | 0.505 | 0.07 (-0.04, 0.17) | 0.213 | 0.387 |
| mCPP | 0.02 (-0.06, 0.10) | 0.588 | 0.854 | 0.05 (-0.03, 0.14) | 0.242 | 0.798 | 0.05 (-0.05, 0.16) | 0.324 | 0.505 | 0.07 (-0.04, 0.17) | 0.213 | 0.387 |
| mBzP | 0.02 (-0.04, 0.06) | 0.328 | 0.854 | 0.07 (0.02, 0.11) | 0.003* | 0.057 | 0.03 (-0.03, 0.08) | 0.300 | 0.505 | 0.02 (-0.04, 0.07) | 0.543 | 0.776 |
| mCIOP | -0.01 (-0.09, 0.07) | 0.840 | 0.913 | -0.04 (-0.13, 0.05) | 0.356 | 0.798 | -0.05 (-0.15, 0.05) | 0.328 | 0.505 | -0.04 (-0.14, 0.06) | 0.453 | 0.696 |
| mCINP | 0.01 (-0.07, 0.08) | 0.707 | 0.913 | 0.02 (-0.06, 0.10) | 0.588 | 0.798 | 0.02 (-0.07, 0.11) | 0.660 | 0.774 | 0.00 (-0.09, 0.09) | 0.962 | 0.962 |
| Values are obtained from linear mixed-effect models that include and interaction term of the exposure with the specific time points. Values represent the SDS change in weight (95% confidence interval (CI)) per natural log increase in the chemical exposure for each time point. Outcome SDs are sex-specific z-scores for age according to World Health Organization growth charts. Estimates are derived from linear mixed models including an interaction term of the exposure with each time point. Model is adjusted for race, education, marital status, insurance, parity, body mass index, alcohol and tabacco use. BP total bipshenol; BPA bisphenol A; BPS bisphenol S; PA phthalic acid; LMW low molecular weight phthalate; mEP mono-ethyl phthalate; mnBP mono-n-butyl phthalate; mIBP mono-isobutyl phthalate; HMW high molecular weight phthalate; DEHP di-(2-ethylhexyl) phthalate; mECPP mono-(2-ethyl-5-carboxypentyl) phthalate; mCMHP mono-(2-carboxymethyl) phthalate; mEHHP mono-(2-ethyl-5-hydroxyhexyl) phthalate; mEOHP mono-2(ethyl-5-oxohexyl) phthalate; mEHP mono-(2-ethylhexyl) phthalate; DNOP di-*n*-octyl phthalate; mCPP mono-(3-carboxypropyl) phthalate; mBzP mono-benzyl phthalate; mCIOP mono-(carboxyisooctyl) phthalate; mCINP mono-(carboxyisononyl) phthalate. FDR False Discovery Rate *Nominal significant p-value <0.05 | | | | | | | | | | | | |

| **Table S9.** Effect of exposures on early childhood body mass index at each time point, adjusted model (n = 672) | | | | | | | | | | | | |
| --- | --- | --- | --- | --- | --- | --- | --- | --- | --- | --- | --- | --- |
|  | **1 year** | | | **2 year** | | | **3 year** | | | **4 year** | | |
| **Chemical** | **Estimate (95% CI)** | **Nominal p-value** | **FDR P-value** | **Estimate (95% CI)** | **Nominal p-value** | **FDR P-value** | **Estimate (95% CI)** | **Nominal p-value** | **FDR P-value** | **Estimate (95% CI)** | **Nominal p-value** | **FDR P-value** |
| BP | 0.10 (-0.03, 0.22) | 0.137 | 0.687 | -0.05 (-0.21, 0.11) | 0.515 | 0.701 | -0.04 (-0.24, 0.15) | 0.654 | 0.860 | -0.01 (-0.19, 0.18) | 0.934 | 0.948 |
| BPA | 0.10 (0.00, 0.21) | 0.057 | 0.568 | -0.10 (-0.24, 0.03) | 0.120 | 0.571 | 0.01 (-0.15, 0.17) | 0.927 | 0.927 | 0.04 (-0.12, 0.20) | 0.602 | 0.916 |
| BPS | 0.02 (-0.08, 0.12) | 0.729 | 0.971 | 0.06 (-0.07, 0.19) | 0.347 | 0.571 | -0.02 (-0.18, 0.14) | 0.821 | 0.864 | -0.01 (-0.16, 0.14) | 0.896 | 0.948 |
| PA | 0.06 (-0.04, 0.16) | 0.257 | 0.788 | 0.08 (-0.04, 0.20) | 0.203 | 0.571 | 0.10 (-0.05, 0.26) | 0.191 | 0.423 | 0.01 (-0.15, 0.16) | 0.948 | 0.948 |
| LMW | -0.08 (-0.19, 0.03) | 0.172 | 0.687 | -0.02 (-0.16, 0.12) | 0.796 | 0.838 | 0.05 (-0.10, 0.21) | 0.500 | 0.820 | 0.01 (-0.16, 0.17) | 0.948 | 0.948 |
| mEP | -0.04 (-0.13, 0.05) | 0.430 | 0.863 | -0.03 (-0.14, 0.09) | 0.632 | 0.743 | 0.04 (-0.09, 0.17) | 0.507 | 0.820 | -0.03 (-0.17, 0.10) | 0.641 | 0.916 |
| mBP | -0.06 (-0.18, 0.05) | 0.276 | 0.788 | 0.01 (-0.14, 0.15) | 0.940 | 0.940 | -0.02 (-0.19, 0.15) | 0.814 | 0.864 | 0.05 (-0.11, 0.21) | 0.564 | 0.916 |
| mIBP | -0.08 (-0.19, 0.03) | 0.144 | 0.687 | 0.06 (-0.07, 0.19) | 0.371 | 0.571 | 0.03 (-0.15, 0.22) | 0.735 | 0.864 | 0.06 (-0.12, 0.23) | 0.525 | 0.916 |
| HMW | 0.00 (-0.13, 0.13) | 0.951 | 0.973 | 0.08 (-0.08, 0.24) | 0.329 | 0.571 | 0.15 (-0.03, 0.33) | 0.097 | 0.295 | 0.24 (0.06, 0.42) | 0.010* | 0.040* |
| DEHP | 0.00 (-0.12, 0.13) | 0.945 | 0.973 | 0.08 (-0.07, 0.24) | 0.288 | 0.571 | 0.16 (-0.01, 0.34) | 0.072 | 0.294 | 0.24 (0.06, 0.41) | 0.009* | 0.040* |
| mECPP | 0.02 (-0.11, 0.15) | 0.811 | 0.973 | 0.08 (-0.08, 0.24) | 0.354 | 0.571 | 0.15 (-0.03, 0.34) | 0.103 | 0.295 | 0.26 (0.07, 0.44) | 0.006* | 0.040* |
| mCMHP | 0.02 (-0.06, 0.11) | 0.561 | 0.863 | 0.06 (-0.04, 0.17) | 0.249 | 0.571 | 0.17 (0.05, 0.30) | 0.007* | 0.132 | 0.14 (0.02, 0.26) | 0.027* | 0.078 |
| mEHHP | -0.03 (-0.15, 0.10) | 0.699 | 0.971 | 0.08 (-0.07, 0.24) | 0.299 | 0.571 | 0.17 (-0.02 ,0.35) | 0.074 | 0.294 | 0.23 (0.06, 0.41) | 0.010* | 0.040* |
| mEOHP | 0.00 (-0.12, 0.13) | 0.968 | 0.973 | 0.09 (-0.06, 0.25) | 0.239 | 0.571 | 0.16 (-0.01, 0.34) | 0.073 | 0.294 | 0.23 (0.06, 0.41) | 0.008* | 0.040* |
| MEHP | 0.03 (-0.06, 0.12) | 0.528 | 0.863 | 0.06 (-0.05, 0.17) | 0.260 | 0.571 | 0.09 (-0.03, 0.21) | 0.136 | 0.340 | 0.15 (0.03, 0.27) | 0.018* | 0.060 |
| DNOP | 0.04 (-0.07, 0.14) | 0.504 | 0.863 | -0.04 (-0.17, 0.09) | 0.561 | 0.701 | -0.03 (-0.20, 0.13) | 0.688 | 0.860 | 0.02 (-0.14, 0.17) | 0.832 | 0.948 |
| mCPP | 0.04 (-0.07, 0.14) | 0.504 | 0.863 | -0.04 (-0.17, 0.09) | 0.561 | 0.701 | -0.03 (-0.20, 0.13) | 0.688 | 0.860 | 0.02 (-0.14, 0.17) | 0.832 | 0.948 |
| mBzP | 0.00 (-0.06, 0.05) | 0.973 | 0.973 | 0.05 (-0.01, 0.12) | 0.122 | 0.571 | 0.03 (-0.06, 0.11) | 0.533 | 0.820 | -0.02 (-0.10, 0.06) | 0.567 | 0.916 |
| mCIOP | 0.03 (-0.07, 0.14) | 0.551 | 0.863 | -0.07 (-0.20, 0.06) | 0.272 | 0.571 | -0.16 (-0.31, 0.00) | 0.048* | 0.294 | -0.04 (-0.20, 0.12) | 0.623 | 0.916 |
| mCINP | 0.11 (0.01, 0.20) | 0.026 | 0.524 | 0.02 (-0.10, 0.14) | 0.763 | 0.838 | -0.05 (-0.18, 0.10) | 0.493 | 0.820 | -0.08 (-0.21, 0.06) | 0.277 | 0.693 |
| Values are obtained from linear mixed-effect models that include and interaction term of the exposure with the specific time points. Values represent the SDS change in body mass index (95% confidence interval (CI)) per natural log increase in the chemical exposure for each time point. Outcome SDs are sex-specific z-scores for age according to World Health Organization growth charts. Estimates are derived from linear mixed models including an interaction term of the exposure with each time point. Model is adjusted for race, education, marital status, insurance, parity, body mass index, alcohol and tabacco use. BP total bipshenol; BPA bisphenol A; BPS bisphenol S; PA phthalic acid; LMW low molecular weight phthalate; mEP mono-ethyl phthalate; mnBP mono-n-butyl phthalate; mIBP mono-isobutyl phthalate; HMW high molecular weight phthalate; DEHP di-(2-ethylhexyl) phthalate; mECPP mono-(2-ethyl-5-carboxypentyl) phthalate; mCMHP mono-(2-carboxymethyl) phthalate; mEHHP mono-(2-ethyl-5-hydroxyhexyl) phthalate; mEOHP mono-2(ethyl-5-oxohexyl) phthalate; mEHP mono-(2-ethylhexyl) phthalate; DNOP di-*n*-octyl phthalate; mCPP mono-(3-carboxypropyl) phthalate; mBzP mono-benzyl phthalate; mCIOP mono-(carboxyisooctyl) phthalate; mCINP mono-(carboxyisononyl) phthalate. FDR False Discovery Rate *Nominal significant p-value <0.05 | | | | | | | | | | | | |

| **Table S10.** Effect of exposures on early childhood triceps skinfold at each time point, adjusted model (n = 558) | | | | | | | | | | | | |
| --- | --- | --- | --- | --- | --- | --- | --- | --- | --- | --- | --- | --- |
|  | **1 year** | | | **2 year** | | | **3 year** | | | **4 year** | | |
| **Chemical** | **Estimate (95% CI)** | **Nominal p-value** | **FDR P-value** | **Estimate (95% CI)** | **Nominal p-value** | **FDR P-value** | **Estimate (95% CI)** | **Nominal p-value** | **FDR P-value** | **Estimate (95% CI)** | **Nominal p-value** | **FDR P-value** |
| BP | 0.10 (-0.03, 0.24) | 0.148 | 0.421 | -0.14 (-0.32, 0.04) | 0.126 | 0.419 | 0.10 (-0.39, 0.56) | 0.716 | 0.978 | -0.10 (-0.39, 0.18) | 0.476 | 0.857 |
| BPA | 0.04 (-0.07, 0.15) | 0.474 | 0.593 | -0.06 (-0.21, 0.09) | 0.413 | 0.583 | 0.04 (-0.35, 0.44) | 0.832 | 0.978 | 0.02 (-0.22, 0.26) | 0.852 | 0.857 |
| BPS | 0.08 (-0.03, 0.19) | 0.142 | 0.421 | -0.09 (-0.24, 0.05) | 0.211 | 0.423 | 0.09 (-0.36, 0.55) | 0.686 | 0.978 | -0.16 (-0.37, 0.05) | 0.129 | 0.857 |
| PA | -0.03 (-0.14, 0.07) | 0.540 | 0.600 | -0.02 (-0.15, 0.11) | 0.756 | 0.799 | -0.10 (-0.51, 0.28) | 0.574 | 0.978 | -0.11 (-0.35, 0.13) | 0.353 | 0.857 |
| LMW | -0.09 (-0.22, 0.03) | 0.129 | 0.421 | 0.07 (-0.09, 0.24) | 0.396 | 0.583 | 0.00 (-0.30, 0.31) | 0.978 | 0.983 | 0.12 (-0.13, 0.36) | 0.359 | 0.857 |
| mEP | -0.06 (-0.16, 0.04) | 0.233 | 0.484 | 0.02 (-0.11, 0.16) | 0.759 | 0.799 | 0.00 (-0.30, 0.29) | 0.983 | 0.983 | 0.04 (-0.15, 0.24) | 0.669 | 0.857 |
| mBP | -0.13 (-0.26, 0.00)* | 0.045 | 0.421 | 0.14 (-0.02, 0.31) | 0.090 | 0.419 | 0.11 (-0.30, 0.51) | 0.619 | 0.978 | 0.09 (-0.14, 0.32) | 0.457 | 0.857 |
| mIBP | -0.05 (-0.17, 0.06) | 0.363 | 0.484 | 0.12 (-0.02, 0.27) | 0.091 | 0.419 | -0.08 (-0.45, 0.29) | 0.676 | 0.978 | 0.23 (0.01, 0.46)* | 0.044 | 0.857 |
| HMW | 0.07 (-0.07, 0.20) | 0.351 | 0.484 | 0.13 (-0.06, 0.32) | 0.189 | 0.419 | -0.11 (-0.45, 0.26) | 0.619 | 0.978 | -0.07 (-0.36, 0.21) | 0.606 | 0.857 |
| DEHP | 0.06 (-0.07, 0.20) | 0.357 | 0.484 | 0.13 (-0.06, 0.31) | 0.188 | 0.419 | -0.09 (-0.43, 0.28) | 0.674 | 0.978 | -0.07 (-0.35, 0.20) | 0.603 | 0.857 |
| mECPP | 0.07 (-0.07, 0.21) | 0.330 | 0.484 | 0.15 (-0.04, 0.35) | 0.123 | 0.419 | -0.05 (-0.40, 0.30) | 0.799 | 0.978 | 0.03 (-0.27, 0.33) | 0.840 | 0.857 |
| mCMHP | 0.02 (-0.06, 0.11) | 0.592 | 0.623 | 0.09 (-0.03, 0.21) | 0.160 | 0.419 | -0.10 (-0.55, 0.29) | 0.552 | 0.978 | -0.04 (-0.23, 0.15) | 0.684 | 0.857 |
| mEHHP | 0.03 (-0.10, 0.17) | 0.651 | 0.651 | 0.15 (-0.03, 0.34) | 0.105 | 0.419 | -0.07 (-0.31, 0.29) | 0.736 | 0.978 | -0.03 (-0.30, 0.24) | 0.814 | 0.857 |
| mEOHP | 0.07 (-0.07, 0.20) | 0.318 | 0.484 | 0.10 (-0.08, 0.29) | 0.276 | 0.485 | -0.12 (-0.46, 0.26) | 0.598 | 0.978 | -0.05 (-0.32, 0.22) | 0.735 | 0.857 |
| MEHP | 0.03 (-0.06, 0.12) | 0.526 | 0.600 | 0.09 (-0.04, 0.22) | 0.178 | 0.419 | 0.04 (-0.34, 0.42) | 0.819 | 0.978 | -0.10 (-0.28, 0.09) | 0.318 | 0.857 |
| DNOP | 0.05 (-0.06, 0.16) | 0.353 | 0.484 | 0.04 (-0.11, 0.18) | 0.623 | 0.733 | -0.28 (-0.75, 0.14) | 0.186 | 0.978 | -0.13 (-0.35, 0.10) | 0.262 | 0.857 |
| mCPP | 0.05 (-0.06, 0.16) | 0.353 | 0.484 | 0.04 (-0.11, 0.18) | 0.623 | 0.733 | -0.28 (-0.75, 0.14) | 0.186 | 0.978 | -0.13 (-0.35, 0.10) | 0.262 | 0.857 |
| mBzP | -0.05 (-0.10, 0.01) | 0.119 | 0.421 | 0.03 (-0.05, 0.11) | 0.437 | 0.583 | -0.00 (-0.49, 0.48) | 0.973 | 0.983 | -0.01 (-0.12, 0.10) | 0.857 | 0.857 |
| mCIOP | 0.09 (-0.02, 0.19) | 0.125 | 0.421 | -0.01 (-0.16, 0.13) | 0.865 | 0.865 | -0.17 (-0.57, 0.21) | 0.368 | 0.978 | -0.04 (-0.24, 0.17) | 0.733 | 0.857 |
| mCINP | 0.08 (-0.02, 0.19) | 0.111 | 0.421 | -0.07 (-0.21, 0.06) | 0.291 | 0.485 | -0.04 (-0.40, 0.30) | 0.786 | 0.978 | -0.07 (-0.27, 0.13) | 0.489 | 0.857 |
| Values are obtained from linear mixed-effect models that include and interaction term of the exposure with the specific time points. Values represent the SDS change in triceps skinfold (95% confidence interval (CI)) per natural log increase in the chemical exposure for each time point. Outcome SDs are sex-specific z-scores for age according to World Health Organization growth charts. Estimates are derived from linear mixed models including an interaction term of the exposure with each time point. Model is adjusted for race, education, marital status, insurance, parity, body mass index, alcohol and tabacco use. BP total bipshenol; BPA bisphenol A; BPS bisphenol S; PA phthalic acid; LMW low molecular weight phthalate; mEP mono-ethyl phthalate; mnBP mono-n-butyl phthalate; mIBP mono-isobutyl phthalate; HMW high molecular weight phthalate; DEHP di-(2-ethylhexyl) phthalate; mECPP mono-(2-ethyl-5-carboxypentyl) phthalate; mCMHP mono-(2-carboxymethyl) phthalate; mEHHP mono-(2-ethyl-5-hydroxyhexyl) phthalate; mEOHP mono-2(ethyl-5-oxohexyl) phthalate; mEHP mono-(2-ethylhexyl) phthalate; DNOP di-*n*-octyl phthalate; mCPP mono-(3-carboxypropyl) phthalate; mBzP mono-benzyl phthalate; mCIOP mono-(carboxyisooctyl) phthalate; mCINP mono-(carboxyisononyl) phthalate. FDR False Discovery Rate. *Nominal significant p-value <0.05 | | | | | | | | | | | | |

| **Table S11.** Effect of exposures on early childhood subscapular skinfold at each time point, adjusted model (n = 415) | | | | | | | | | | | | |
| --- | --- | --- | --- | --- | --- | --- | --- | --- | --- | --- | --- | --- |
|  | **1 year** | | | **2 year** | | | **3 year** | | | **4 year** | | |
| **Chemical** | **Estimate (95% CI)** | **Nominal p-value** | **FDR P-value** | **Estimate (95% CI)** | **Nominal p-value** | **FDR P-value** | **Estimate (95% CI)** | **Nominal p-value** | **FDR P-value** | **Estimate (95% CI)** | **Nominal p-value** | **FDR P-value** |
| BP | -0.17 (-0.39, 0.06) | 0.142 | 0.801 | 0.14 (-0.11, 0.40) | 0.265 | 0.648 | 0.03 (-0.47, 0.53) | 0.914 | 0.914 | 0.16 (-0.15, 0.47) | 0.344 | 0.993 |
| BPA | -0.05 (-0.22, 0.12) | 0.559 | 0.931 | 0.06 (-0.14, 0.25) | 0.578 | 0.648 | 0.07 (-0.29, 0.43) | 0.689 | 0.911 | 0.19 (-0.07, 0.44) | 0.142 | 0.993 |
| BPS | -0.12 (-0.29, 0.05) | 0.164 | 0.801 | 0.10 (-0.10, 0.30) | 0.332 | 0.648 | -0.05 (-0.44, 0.34) | 0.789 | 0.911 | 0.00 (-0.26, 0.24) | 0.990 | 0.993 |
| PA | -0.11 (-0.29, 0.08) | 0.276 | 0.801 | 0.06 (-0.14, 0.26) | 0.583 | 0.648 | -0.11 (-0.36, 0.21) | 0.499 | 0.895 | -0.08 (-0.42, 0.20) | 0.570 | 0.993 |
| LMW | -0.15 (-0.33, 0.04) | 0.124 | 0.801 | 0.08 (-0.12, 0.29) | 0.430 | 0.648 | -0.06 (-0.27, 0.24) | 0.704 | 0.911 | 0.03 (-0.24, 0.30) | 0.857 | 0.993 |
| mEP | -0.12 (-0.27, 0.03) | 0.123 | 0.801 | 0.07 (-0.11, 0.24) | 0.459 | 0.648 | -0.03 (-0.43, 0.21) | 0.824 | 0.911 | 0.00 (-0.26, 0.22) | 0.993 | 0.993 |
| mBP | -0.11 (-0.31, 0.09) | 0.281 | 0.801 | 0.12 (-0.10, 0.35) | 0.276 | 0.648 | -0.03 (-0.62, 0.36) | 0.866 | 0.911 | 0.08 (-0.19, 0.36) | 0.566 | 0.993 |
| mIBP | 0.03 (-0.18, 0.24) | 0.803 | 0.931 | -0.02 (-0.24, 0.21) | 0.890 | 0.890 | -0.25 (-0.54, 0.12) | 0.184 | 0.895 | 0.00 (-0.27, 0.29) | 0.984 | 0.993 |
| HMW | -0.02 (-0.22, 0.18) | 0.852 | 0.931 | 0.20 (-0.04, 0.43) | 0.103 | 0.557 | -0.14 (-0.51, 0.26) | 0.506 | 0.895 | 0.10 (-0.18, 0.41) | 0.533 | 0.993 |
| DEHP | -0.02 (-0.22, 0.19) | 0.884 | 0.931 | 0.19 (-0.04, 0.43) | 0.111 | 0.557 | -0.11 (-0.51, 0.28) | 0.573 | 0.895 | 0.10 (-0.18, 0.40) | 0.539 | 0.993 |
| mECPP | 0.01 (-0.19, 0.21) | 0.912 | 0.931 | 0.22 (-0.01, 0.46) | 0.068 | 0.557 | -0.11 (-0.51, 0.28) | 0.582 | 0.895 | 0.44 (-0.18, 0.43) | 0.499 | 0.993 |
| mCMHP | -0.08 (-0.22, 0.06) | 0.276 | 0.801 | 0.19 (0.03, 0.35)* | 0.021 | 0.427 | 0.03 (-0.28, 0.33) | 0.860 | 0.911 | 0.20 (-0.02, 0.41) | 0.070 | 0.993 |
| mEHHP | -0.01 (-0.21, 0.19) | 0.914 | 0.931 | 0.17 (-0.06, 0.40) | 0.154 | 0.614 | -0.14 (-0.54, 0.25) | 0.491 | 0.895 | 0.10 (-0.16, 0.40) | 0.503 | 0.993 |
| mEOHP | 0.02 (-0.18, 0.23) | 0.822 | 0.931 | 0.13 (-0.11, 0.37) | 0.283 | 0.648 | -0.16 (-0.56, 0.25) | 0.456 | 0.895 | 0.03 (-0.24, 0.33) | 0.859 | 0.993 |
| MEHP | 0.01 (-0.14, 0.15) | 0.931 | 0.931 | 0.07 (-0.10, 0.23) | 0.416 | 0.648 | -0.12 (-0.41, 0.17) | 0.431 | 0.895 | 0.09 (-0.15, 0.30) | 0.396 | 0.993 |
| DNOP | -0.01 (-0.22, 0.19) | 0.896 | 0.931 | 0.07 (-0.15, 0.30) | 0.520 | 0.648 | -0.43 (-0.82, -0.03) | 0.039* | 0.386 | -0.02 (-0.32, 0.25) | 0.876 | 0.993 |
| mCPP | -0.01 (-0.22, 0.19) | 0.896 | 0.931 | 0.07 (-0.15, 0.30) | 0.520 | 0.648 | -0.43 (-0.82, -0.03) | 0.039* | 0.386 | -0.02 (-0.32, 0.25) | 0.876 | 0.993 |
| mBzP | -0.03 (-0.13, 0.08) | 0.595 | 0.931 | 0.04 (-0.07, 0.16) | 0.449 | 0.648 | -0.08 (-0.31, 0.14) | 0.489 | 0.895 | -0.01 (-0.30, 0.13) | 0.944 | 0.993 |
| mCIOP | 0.08 (-0.10, 0.25) | 0.405 | 0.931 | 0.03 (-0.17, 0.23) | 0.763 | 0.803 | -0.33 (-0.67, 0.01) | 0.064 | 0.428 | -0.06 (-0.33, 0.18) | 0.605 | 0.993 |
| mCINP | -0.04 (-0.21, 0.12) | 0.626 | 0.931 | 0.05 (-0.13, 0.24) | 0.582 | 0.648 | -0.18 (-0.46, 0.11) | 0.234 | 0.895 | 0.05 (-0.22, 0.28) | 0.704 | 0.993 |
| Values are obtained from linear mixed-effect models that include and interaction term of the exposure with the specific time points. Values represent the SDS change in subscapular skinfold (95% confidence interval (CI)) per natural log increase in the chemical exposure for each time point. Estimates are derived from linear mixed models including an interaction term of the exposure with each time point. Model is adjusted for race, education, marital status, insurance, parity, body mass index, alcohol and tabacco use. BP total bipshenol; BPA bisphenol A; BPS bisphenol S; PA phthalic acid; LMW low molecular weight phthalate; mEP mono-ethyl phthalate; mnBP mono-n-butyl phthalate; mIBP mono-isobutyl phthalate; HMW high molecular weight phthalate; DEHP di-(2-ethylhexyl) phthalate; mECPP mono-(2-ethyl-5-carboxypentyl) phthalate; mCMHP mono-(2-carboxymethyl) phthalate; mEHHP mono-(2-ethyl-5-hydroxyhexyl) phthalate; mEOHP mono-2(ethyl-5-oxohexyl) phthalate; mEHP mono-(2-ethylhexyl) phthalate; DNOP di-*n*-octyl phthalate; mCPP mono-(3-carboxypropyl) phthalate; mBzP mono-benzyl phthalate; mCIOP mono-(carboxyisooctyl) phthalate; mCINP mono-(carboxyisononyl) phthalate. FDR False Discovery Rate. *Nominal significant p-value <0.05 | | | | | | | | | | | | |

| **Table S12.** Associations of the individual and grouped metabolites with childhood adiposity outcomes between birth and 4 years in boys, adjusted model | | | | | | | | | | | | |
| --- | --- | --- | --- | --- | --- | --- | --- | --- | --- | --- | --- | --- |
|  | **Weight SDS (n = 576)** | | | **Body mass index SDS (n = 351)** | | | **Triceps skinfold SDS (n = 296)** | | | **Subscapular skinfold SDS (n = 221)** | | |
| **Chemical** | **Estimate (95% CI)** | **Nominal p-value** | **FDR P-value** | **Estimate (95% CI)** | **Nominal p-value** | **FDR P-value** | **Estimate (95% CI)** | **Nominal p-value** | **FDR P-value** | **Estimate (95% CI)** | **Nominal p-value** | **FDR P-value** |
| BP | 0.06 (-0.03, 0.14) | 0.191 | 0.614 | 0.06 (-0.03, 0.14) | 0.193 | 0.639 | -0.02 (-0.18, 0.13) | 0.770 | 0.887 | -0.09 (-0.27, 0.09) | 0.326 | 0.655 |
| BPA | 0.04 (-0.03, 0.12) | 0.232 | 0.614 | 0.04 (-0.03, 0.12) | 0.249 | 0.639 | -0.03 (-0.16, 0.10) | 0.670 | 0.887 | -0.01 (-0.15, 0.13) | 0.900 | 0.900 |
| BPS | 0.03 (-0.03, 0.10) | 0.327 | 0.614 | 0.03 (-0.03, 0.10) | 0.320 | 0.639 | -0.02 (-0.15, 0.10) | 0.737 | 0.887 | -0.12 (-0.27, 0.04) | 0.149 | 0.655 |
| PA | 0.08 (0.01, 0.15)* | 0.028 | 0.381 | 0.08 (0.01, 0.15)* | 0.027 | 0.404 | -0.09 (-0.22, 0.04) | 0.189 | 0.887 | -0.10 (-0.24, 0.05) | 0.216 | 0.655 |
| LMW | 0.00 (-0.08, 0.08) | 1.000 | 1.000 | 0.00 (-0.08, 0.08) | 0.983 | 0.983 | -0.10 (-0.24, 0.05) | 0.191 | 0.887 | -0.12 (-0.28, 0.04) | 0.155 | 0.655 |
| mEP | 0.00 (-0.06, 0.07) | 0.943 | 0.993 | 0.00 (-0.06, 0.06) | 0.978 | 0.983 | -0.08 (-0.19, 0.04) | 0.209 | 0.887 | -0.09 (-0.22, 0.04) | 0.200 | 0.655 |
| mBP | -0.01 (-0.08, 0.07) | 0.843 | 0.948 | -0.01 (-0.08, 0.07) | 0.865 | 0.983 | -0.12 (-0.26, 0.03) | 0.113 | 0.887 | -0.08 (-0.25, 0.08) | 0.327 | 0.655 |
| mIBP | -0.01 (-0.08, 0.06) | 0.730 | 0.912 | -0.01 (-0.08, 0.06) | 0.750 | 0.938 | 0.01 (-0.11, 0.14) | 0.842 | 0.887 | -0.02 (-0.16, 0.12) | 0.770 | 0.811 |
| HMW | 0.04 (-0.05, 0.13) | 0.359 | 0.614 | 0.04 (-0.05, 0.13) | 0.377 | 0.639 | 0.03 (-0.14, 0.20) | 0.732 | 0.887 | 0.07 (-0.13, 0.26) | 0.524 | 0.655 |
| DEHP | 0.04 (-0.05, 0.13) | 0.368 | 0.614 | 0.04 (-0.05, 0.13) | 0.386 | 0.639 | 0.04 (-0.13, 0.20) | 0.674 | 0.887 | 0.07 (-0.12, 0.27) | 0.476 | 0.655 |
| mECPP | 0.04 (-0.05, 0.13) | 0.390 | 0.614 | 0.04 (-0.05, 0.13) | 0.389 | 0.639 | 0.06 (-0.11, 0.23) | 0.468 | 0.887 | 0.09 (-0.11, 0.29) | 0.379 | 0.655 |
| mCMHP | 0.06 (0.00, 0.12)* | 0.043 | 0.381 | 0.06 (0.00, 0.12)* | 0.047 | 0.404 | 0.03 (-0.08, 0.14) | 0.577 | 0.887 | 0.04 (-0.09, 0.17) | 0.523 | 0.655 |
| mEHHP | 0.04 (-0.05, 0.13) | 0.394 | 0.614 | 0.04 (-0.05, 0.12) | 0.414 | 0.639 | 0.02 (-0.14, 0.19) | 0.801 | 0.887 | 0.06 (-0.13, 0.25) | 0.558 | 0.657 |
| mEOHP | 0.04 (-0.05, 0.12) | 0.399 | 0.614 | 0.04 (-0.05, 0.12) | 0.415 | 0.639 | 0.06 (-0.10, 0.22) | 0.492 | 0.887 | 0.07 (-0.12, 0.26) | 0.485 | 0.655 |
| MEHP | 0.02 (-0.04, 0.08) | 0.572 | 0.762 | 0.01 (-0.04, 0.07) | 0.625 | 0.834 | 0.00 (-0.11, 0.11) | 0.975 | 0.975 | 0.06 (-0.07, 0.18) | 0.400 | 0.655 |
| DNOP | 0.05 (-0.03, 0.12) | 0.202 | 0.614 | 0.05 (-0.03, 0.12) | 0.209 | 0.639 | -0.07 (-0.20, 0.07) | 0.343 | 0.887 | -0.07 (-0.22, 0.09) | 0.430 | 0.655 |
| mCPP | 0.05 (-0.03, 0.12) | 0.202 | 0.614 | 0.05 (-0.03, 0.12) | 0.209 | 0.639 | -0.07 (-0.20, 0.07) | 0.343 | 0.887 | -0.07 (-0.22, 0.09) | 0.430 | 0.655 |
| mBzP | 0.00 (-0.03, 0.04) | 0.853 | 0.948 | 0.00 (-0.03, 0.04) | 0.899 | 0.983 | -0.03 (-0.09, 0.04) | 0.450 | 0.887 | -0.04 (-0.11, 0.04) | 0.362 | 0.655 |
| mCIOP | 0.02 (-0.05, 0.10) | 0.522 | 0.745 | 0.02 (-0.05, 0.10) | 0.559 | 0.799 | -0.02 (-0.16, 0.12) | 0.829 | 0.887 | 0.03 (-0.14, 0.19) | 0.762 | 0.811 |
| mCINP | 0.06 (0.00, 0.13) | 0.057 | 0.381 | 0.06 (0.00, 0.13) | 0.061 | 0.404 | 0.02 (-0.11, 0.14) | 0.795 | 0.887 | -0.06 (-0.20, 0.08) | 0.394 | 0.655 |
| Values represent the SDS change in adiposity outcomes (95% confidence interval (CI)) per natural log increase in the chemical exposure. Outcome SDs are sex-specific z-scores for age according to World Health Organization growth charts. BP total bipshenol; BPA bisphenol A; BPS bisphenol S; PA phthalic acid; LMW low molecular weight phthalate; mEP mono-ethyl phthalate; mnBP mono-n-butyl phthalate; mIBP mono-isobutyl phthalate; HMW high molecular weight phthalate; DEHP di-(2-ethylhexyl) phthalate; mECPP mono-(2-ethyl-5-carboxypentyl) phthalate; mCMHP mono-(2-carboxymethyl) phthalate; mEHHP mono-(2-ethyl-5-hydroxyhexyl) phthalate; mEOHP mono-2(ethyl-5-oxohexyl) phthalate; mEHP mono-(2-ethylhexyl) phthalate; DNOP di-*n*-octyl phthalate; mCPP mono-(3-carboxypropyl) phthalate; mBzP mono-benzyl phthalate; mCIOP mono-(carboxyisooctyl) phthalate; mCINP mono-(carboxyisononyl) phthalate. FDR False Discovery Rate. *Nominal significant p-value <0.05 | | | | | | | | | | | | |

| **Table S13.** Associations of the individual and grouped metabolites with childhood adiposity outcomes between birth and 4 years in girls, adjusted model | | | | | | | | | | | | |
| --- | --- | --- | --- | --- | --- | --- | --- | --- | --- | --- | --- | --- |
|  | **Weight SDS (n = 515)** | | | **Body mass index SDS (n = 321)** | | | **Triceps skinfold SDS (n = 262)** | | | **Subscapular skinfold SDS (n = 194)** | | |
| **Chemical** | **Estimate (95% CI)** | **Nominal p-value** | **FDR P-value** | **Estimate (95% CI)** | **Nominal p-value** | **FDR P-value** | **Estimate (95% CI)** | **Nominal p-value** | **FDR P-value** | **Estimate (95% CI)** | **Nominal p-value** | **FDR P-value** |
| BP | 0.06 (-0.03, 0.15) | 0.189 | 0.656 | 0.06 (-0.03, 0.15) | 0.171 | 0.688 | 0.11 (-0.05,0.27) | 0.191 | 0.348 | -0.09 (-0.27, 0.09) | 0.326 | 0.655 |
| BPA | 0.07 (0.00, 0.15) | 0.062 | 0.656 | 0.07 (0.00, 0.15) | 0.057 | 0.688 | 0.06 (-0.07, 0.20) | 0.375 | 0.526 | -0.01 (-0.15, 0.13) | 0.900 | 0.900 |
| BPS | 0.03 (-0.05, 0.10) | 0.473 | 0.859 | 0.03 (-0.04, 0.10) | 0.454 | 0.825 | 0.09 (-0.03, 0.21) | 0.164 | 0.328 | -0.12 (-0.27, 0.04) | 0.149 | 0.655 |
| PA | 0.02 (-0.04, 0.09) | 0.545 | 0.908 | 0.02 (-0.04, 0.09) | 0.520 | 0.867 | -0.01 (-0.12, 0.10) | 0.831 | 0.879 | -0.10 (-0.24, 0.05) | 0.216 | 0.655 |
| LMW | 0.00 (-0.08, 0.08) | 0.997 | 0.997 | 0.00 (-0.08, 0.08) | 0.962 | 0.962 | -0.02 (-0.16, 0.12) | 0.762 | 0.879 | -0.12 (-0.28, 0.04) | 0.155 | 0.655 |
| mEP | 0.00 (-0.06, 0.07) | 0.930 | 0.997 | 0.00 (-0.06, 0.07) | 0.942 | 0.962 | -0.01 (-0.13, 0.10) | 0.843 | 0.879 | -0.09 (-0.22, 0.04) | 0.200 | 0.655 |
| mBP | -0.02 (-0.11, 0.07) | 0.615 | 0.946 | -0.02 (-0.11, 0.07) | 0.727 | 0.962 | -0.05 (-0.20, 0.09) | 0.479 | 0.599 | -0.08 (-0.25, 0.08) | 0.327 | 0.655 |
| mIBP | 0.01 (-0.08, 0.09) | 0.869 | 0.997 | 0.01 (-0.08, 0.09) | 0.825 | 0.962 | 0.01 (-0.12, 0.15) | 0.879 | 0.879 | -0.02 (-0.16, 0.12) | 0.770 | 0.811 |
| HMW | 0.05 (-0.05, 0.15) | 0.304 | 0.656 | 0.05 (-0.04, 0.15) | 0.294 | 0.688 | 0.13 (-0.03, 0.29) | 0.132 | 0.328 | 0.07 (-0.13, 0.26) | 0.524 | 0.655 |
| DEHP | 0.06 (-0.04, 0.15) | 0.263 | 0.656 | 0.06 (-0.04, 0.15) | 0.254 | 0.688 | 0.12 (-0.04, 0.28) | 0.153 | 0.328 | 0.07 (-0.12, 0.27) | 0.476 | 0.655 |
| mECPP | 0.05 (-0.05, 0.15) | 0.305 | 0.656 | 0.05 (-0.05, 0.15) | 0.309 | 0.688 | 0.13 (-0.03, 0.30) | 0.125 | 0.328 | 0.09 (-0.11, 0.29) | 0.379 | 0.655 |
| mCMHP | 0.03 (-0.03, 0.09) | 0.277 | 0.656 | 0.03 (-0.03, 0.09) | 0.263 | 0.688 | 0.04 (-0.06, 0.15) | 0.394 | 0.526 | 0.04 (-0.09, 0.17) | 0.523 | 0.655 |
| mEHHP | 0.07 (-0.03, 0.16) | 0.157 | 0.656 | 0.07 (-0.02, 0.17) | 0.144 | 0.688 | 0.09 (-0.07, 0.25) | 0.264 | 0.407 | 0.06 (-0.13, 0.25) | 0.558 | 0.657 |
| mEOHP | 0.07 (-0.03, 0.16) | 0.171 | 0.656 | 0.07 (-0.03, 0.17) | 0.154 | 0.688 | 0.10 (-0.07, 0.26) | 0.256 | 0.407 | 0.07 (-0.12, 0.26) | 0.485 | 0.655 |
| MEHP | 0.04 (-0.02, 0.11) | 0.182 | 0.656 | 0.04 (-0.02, 0.11) | 0.194 | 0.688 | 0.10 (-0.01, 0.21) | 0.085 | 0.328 | 0.06 (-0.07, 0.18) | 0.400 | 0.655 |
| DNOP | -0.01 (-0.08, 0.07) | 0.857 | 0.997 | 0.00 (-0.08, 0.07) | 0.920 | 0.962 | 0.14 (0.01, 0.26)* | 0.032 | 0.213 | -0.07 (-0.22, 0.09) | 0.430 | 0.655 |
| mCPP | -0.01 (-0.08, 0.07) | 0.857 | 0.997 | 0.00 (-0.08, 0.07) | 0.920 | 0.962 | 0.14 (0.01, 0.26)* | 0.032 | 0.213 | -0.07 (-0.22, 0.09) | 0.430 | 0.655 |
| mBzP | -0.02 (-0.06, 0.02) | 0.328 | 0.656 | -0.02 (-0.06, 0.02) | 0.352 | 0.704 | -0.05 (-0.12, 0.02) | 0.160 | 0.328 | -0.04 (-0.11, 0.04) | 0.362 | 0.655 |
| mCIOP | 0.00 (-0.07, 0.07) | 0.954 | 0.997 | 0.00 (-0.07, 0.08) | 0.927 | 0.962 | 0.15 (0.03, 0.26)* | 0.020 | 0.213 | 0.03 (-0.14, 0.19) | 0.762 | 0.811 |
| mCINP | 0.01 (-0.06, 0.07) | 0.896 | 0.997 | 0.01 (-0.06, 0.07) | 0.873 | 0.962 | 0.10 (-0.02, 0.22) | 0.117 | 0.328 | -0.06 (-0.20, 0.08) | 0.394 | 0.655 |
| Values are obtained from linear mixed-effect models and represent the SDS change in weight (95% confidence interval (CI)) between birth and 4 years per natural log increase in the chemical exposure. Outcome SDs are sex-specific z-scores for age according to World Health Organization growth charts. Model is adjusted for race, education, marital status, insurance, parity, body mass index, alcohol and tabacco use. BP total bipshenol; BPA bisphenol A; BPS bisphenol S; PA phthalic acid; LMW low molecular weight phthalate; mEP mono-ethyl phthalate; mnBP mono-n-butyl phthalate; mIBP mono-isobutyl phthalate; HMW high molecular weight phthalate; DEHP di-(2-ethylhexyl) phthalate; mECPP mono-(2-ethyl-5-carboxypentyl) phthalate; mCMHP mono-(2-carboxymethyl) phthalate; mEHHP mono-(2-ethyl-5-hydroxyhexyl) phthalate; mEOHP mono-2(ethyl-5-oxohexyl) phthalate; mEHP mono-(2-ethylhexyl) phthalate; DNOP di-*n*-octyl phthalate; mCPP mono-(3-carboxypropyl) phthalate; mBzP mono-benzyl phthalate; mCIOP mono-(carboxyisooctyl) phthalate; mCINP mono-(carboxyisononyl) phthalate. FDR False Discovery Rate. | | | | | | | | | | | | |

| **Table S14.** Associations of the individual and grouped metabolites with childhood adiposity outcomes between birth and 2 years, adjusted model | | | | | | | | | | | | |
| --- | --- | --- | --- | --- | --- | --- | --- | --- | --- | --- | --- | --- |
|  | **Weight SDS (n = 1087)** | | | **Body mass index SDS (n = 642)** | | | **Triceps skinfold SDS (n = 549)** | | | **Subscapular skinfold SDS (n = 382)** | | |
| **Chemical** | **Estimate (95% CI)** | **Nominal p-value** | **FDR P-value** | **Estimate (95% CI)** | **Nominal p-value** | **FDR P-value** | **Estimate (95% CI)** | **Nominal p-value** | **FDR P-value** | **Estimate (95% CI)** | **Nominal p-value** | **FDR P-value** |
| BP | 0.06 (0.00, 0.12) | 0.060 | 0.605 | 0.07 (-0.03, 0.17) | 0.170 | 0.509 | 0.04 (-0.07, 0.16) | 0.473 | 0.526 | -0.05 (-0.18, 0.09) | 0.497 | 0.663 |
| BPA | 0.05 (0.00, 0.10) | 0.050 | 0.605 | 0.06 (-0.03, 0.14) | 0.178 | 0.509 | 0.02 (-0.08, 0.11) | 0.759 | 0.799 | 0.00 (-0.10, 0.11) | 0.989 | 0.989 |
| BPS | 0.03 (-0.01, 0.08) | 0.176 | 0.621 | 0.04 (-0.04, 0.12) | 0.293 | 0.512 | 0.05 (-0.05, 0.14) | 0.330 | 0.412 | -0.05 (-0.15, 0.06) | 0.397 | 0.574 |
| PA | 0.04 (-0.01, 0.08) | 0.100 | 0.621 | 0.09 (0.01, 0.17)* | 0.023 | 0.232 | -0.04 (-0.13, 0.05) | 0.391 | 0.460 | -0.05 (-0.15, 0.04) | 0.269 | 0.490 |
| LMW | 0.00 (-0.05, 0.05) | 0.953 | 0.987 | -0.08 (-0.17, 0.01) | 0.096 | 0.509 | -0.06 (-0.17, 0.04) | 0.261 | 0.397 | -0.07 (-0.18, 0.05) | 0.253 | 0.490 |
| mEP | 0.00 (-0.04, 0.04) | 0.967 | 0.987 | -0.04 (-0.12, 0.03) | 0.232 | 0.512 | -0.05 (-0.14, 0.04) | 0.264 | 0.397 | -0.06 (-0.18, 0.03) | 0.205 | 0.455 |
| mBP | 0.00 (-0.06, 0.05) | 0.987 | 0.987 | -0.05 (-0.14, 0.04) | 0.282 | 0.512 | -0.06 (-0.17, 0.05) | 0.298 | 0.397 | 0.00 (-0.16, 0.12) | 0.951 | 0.989 |
| mIBP | 0.00 (-0.06, 0.05) | 0.901 | 0.987 | -0.05 (-0.14, 0.03) | 0.227 | 0.512 | 0.00 (-0.10, 0.10) | 0.966 | 0.966 | 0.02 (-0.12, 0.12) | 0.735 | 0.899 |
| HMW | 0.02 (-0.04, 0.09) | 0.458 | 0.680 | 0.03 (-0.07, 0.14) | 0.528 | 0.661 | 0.11 (-0.01, 0.24) | 0.068 | 0.355 | 0.13 (-0.09, 0.27) | 0.063 | 0.317 |
| DEHP | 0.02 (-0.04, 0.09) | 0.447 | 0.680 | 0.04 (-0.07, 0.14) | 0.491 | 0.661 | 0.11 (-0.01, 0.23) | 0.071 | 0.355 | 0.13 (-0.01, 0.26) | 0.062 | 0.317 |
| mECPP | 0.02 (-0.04, 0.09) | 0.467 | 0.680 | 0.05 (-0.06, 0.15) | 0.374 | 0.575 | 0.12 (0.00, 0.25) | 0.053 | 0.355 | 0.17 (0.00, 0.30)* | 0.019* | 0.317 |
| mCMHP | 0.03 (-0.01, 0.07) | 0.186 | 0.621 | 0.05 (-0.02, 0.11) | 0.178 | 0.509 | 0.05 (-0.02, 0.13) | 0.177 | 0.355 | 0.06 (-0.03, 0.15) | 0.167 | 0.418 |
| mEHHP | 0.03 (-0.03, 0.09) | 0.329 | 0.680 | 0.00 (-0.10, 0.11) | 0.953 | 0.972 | 0.09 (-0.03, 0.21) | 0.137 | 0.355 | 0.12 (-0.01, 0.25) | 0.082 | 0.326 |
| mEOHP | 0.03 (-0.03, 0.09) | 0.386 | 0.680 | 0.04 (-0.07, 0.14) | 0.496 | 0.661 | 0.11 (-0.01, 0.23) | 0.081 | 0.355 | 0.13 (0.00, 0.27) | 0.059 | 0.317 |
| MEHP | 0.02 (-0.03, 0.06) | 0.449 | 0.680 | 0.06 (-0.01, 0.13) | 0.115 | 0.509 | 0.07 (-0.01, 0.15) | 0.113 | 0.355 | 0.07 (-0.02, 0.16) | 0.120 | 0.344 |
| DNOP | 0.02 (-0.03, 0.07) | 0.510 | 0.680 | 0.02 (-0.07, 0.10) | 0.719 | 0.799 | 0.07 (-0.03, 0.17) | 0.164 | 0.355 | 0.05 (-0.06, 0.16) | 0.402 | 0.574 |
| mCPP | 0.02 (-0.03, 0.07) | 0.510 | 0.680 | 0.02 (-0.07, 0.10) | 0.719 | 0.799 | 0.07 (-0.03, 0.17) | 0.164 | 0.355 | 0.05 (-0.06, 0.16) | 0.402 | 0.574 |
| mBzP | -0.01 (-0.03, 0.02) | 0.690 | 0.863 | 0.02 (-0.02, 0.07) | 0.307 | 0.512 | -0.03 (-0.08, 0.02) | 0.207 | 0.377 | 0.01 (-0.05, 0.07) | 0.764 | 0.899 |
| mCIOP | 0.02 (-0.03, 0.07) | 0.391 | 0.680 | 0.00 (-0.09, 0.08) | 0.972 | 0.972 | 0.08 (-0.02, 0.18) | 0.105 | 0.355 | 0.09 (-0.02, 0.20) | 0.098 | 0.326 |
| mCINP | 0.03 (-0.01, 0.08) | 0.129 | 0.621 | 0.11 (0.03, 0.18)* | 0.007 | 0.139 | 0.05 (-0.04, 0.14) | 0.293 | 0.397 | 0.00 (-0.10, 0.10) | 0.962 | 0.989 |
| Values are obtained from linear mixed-effect models and represent the SDS change in weight (95% confidence interval (CI)) between birth and 4 years per natural log increase in the chemical exposure. Outcome SDs are sex-specific z-scores for age according to World Health Organization growth charts. Model is adjusted for race, education, marital status, insurance, parity, body mass index, alcohol and tabacco use. BP total bipshenol; BPA bisphenol A; BPS bisphenol S; PA phthalic acid; LMW low molecular weight phthalate; mEP mono-ethyl phthalate; mnBP mono-n-butyl phthalate; mIBP mono-isobutyl phthalate; HMW high molecular weight phthalate; DEHP di-(2-ethylhexyl) phthalate; mECPP mono-(2-ethyl-5-carboxypentyl) phthalate; mCMHP mono-(2-carboxymethyl) phthalate; mEHHP mono-(2-ethyl-5-hydroxyhexyl) phthalate; mEOHP mono-2(ethyl-5-oxohexyl) phthalate; mEHP mono-(2-ethylhexyl) phthalate; DNOP di-*n*-octyl phthalate; mCPP mono-(3-carboxypropyl) phthalate; mBzP mono-benzyl phthalate; mCIOP mono-(carboxyisooctyl) phthalate; mCINP mono-(carboxyisononyl) phthalate. *Nominal significant p-value <0.05 | | | | | | | | | | | | |

| **Table S15.** Associations of the individual and grouped metabolites with childhood adiposity outcomes between birth and 4 years including inverse probability of censoring weights, adjusted model | | | | | | | | | | | | |
| --- | --- | --- | --- | --- | --- | --- | --- | --- | --- | --- | --- | --- |
|  | **Weight in SDS (n = 1091)** | | | **Body mass index in SDS (n = 672)** | | | **Triceps skinfold in SDS (n = 558)** | | | **Subscapular skinfold in SDS (n = 415)** | | |
| **Chemical** | **Estimate (95% CI)** | **Nominal p-value** | **FDR P-value** | **Estimate (95% CI)** | **Nominal p-value** | **FDR P-value** | **Estimate (95% CI)** | **Nominal p-value** | **FDR P-value** | **Estimate (95% CI)** | **Nominal p-value** | **FDR P-value** |
| BP | 0.06 (0.00, 0.13)* | 0.045 | 0.260 | 0.08 (-0.03, 0.18) | 0.142 | 0.258 | 0.05 (-0.07, 0.16) | 0.433 | 0.488 | -0.06 (-0.18, 0.07) | 0.365 | 0.562 |
| BPA | 0.06 (0.01, 0.12)* | 0.020 | 0.260 | 0.08 (0.00, 0.17) | 0.055 | 0.180 | 0.02 (-0.07, 0.12) | 0.604 | 0.635 | 0.01 (-0.09, 0.12) | 0.781 | 0.940 |
| BPS | 0.03 (-0.02, 0.08) | 0.219 | 0.365 | 0.03 (-0.05, 0.11) | 0.449 | 0.562 | 0.04 (-0.05, 0.12) | 0.439 | 0.488 | -0.08 (-0.18, 0.02) | 0.137 | 0.433 |
| PA | 0.05 (0.00, 0.09) | 0.054 | 0.260 | 0.10 (0.02, 0.18)* | 0.019 | 0.093 | -0.05 (-0.14, 0.03) | 0.227 | 0.365 | -0.08 (-0.18, 0.01) | 0.079 | 0.397 |
| LMW | 0.00 (-0.06, 0.05) | 0.956 | 0.991 | -0.07 (-0.16, 0.02) | 0.108 | 0.217 | -0.06 (-0.16, 0.04) | 0.228 | 0.365 | -0.11 (-0.21, 0.00) | 0.061 | 0.397 |
| mEP | 0.00 (-0.05, 0.05) | 0.991 | 0.991 | -0.04 (-0.12, 0.03) | 0.262 | 0.349 | -0.05 (-0.13, 0.03) | 0.230 | 0.365 | -0.09 (-0.18, 0.00)* | 0.048 | 0.397 |
| mBP | -0.01 (-0.07, 0.05) | 0.791 | 0.931 | -0.06 (-0.15, 0.04) | 0.229 | 0.349 | -0.07 (-0.17, 0.04) | 0.202 | 0.365 | -0.03 (-0.15, 0.08) | 0.584 | 0.834 |
| mIBP | 0.00 (-0.06, 0.06) | 0.988 | 0.991 | -0.05 (-0.14, 0.04) | 0.249 | 0.349 | 0.01 (-0.08, 0.11) | 0.777 | 0.777 | 0.01 (-0.10, 0.11) | 0.918 | 0.970 |
| HMW | 0.05 (-0.01, 0.12) | 0.127 | 0.260 | 0.09 (-0.01, 0.20) | 0.089 | 0.197 | 0.09 (-0.03, 0.21) | 0.127 | 0.365 | 0.08 (-0.05, 0.21) | 0.214 | 0.433 |
| DEHP | 0.05 (-0.01, 0.12) | 0.116 | 0.260 | 0.09 (-0.01, 0.20) | 0.072 | 0.180 | 0.09 (-0.03, 0.21) | 0.127 | 0.365 | 0.08 (-0.04, 0.21) | 0.197 | 0.433 |
| mECPP | 0.05 (-0.02, 0.12) | 0.130 | 0.260 | 0.10 (0.00, 0.21) | 0.058 | 0.180 | 0.11 (-0.01, 0.23) | 0.068 | 0.365 | 0.12 (-0.01, 0.25) | 0.073 | 0.397 |
| mCMHP | 0.05 (0.01, 0.09)* | 0.026 | 0.260 | 0.09 (0.02, 0.16)* | 0.011 | 0.093 | 0.04 (-0.03, 0.12) | 0.255 | 0.365 | 0.06 (-0.03, 0.14) | 0.192 | 0.433 |
| mEHHP | 0.06 (-0.01, 0.12) | 0.080 | 0.260 | 0.07 (-0.04, 0.17) | 0.214 | 0.349 | 0.07 (-0.04, 0.19) | 0.222 | 0.365 | 0.08 (-0.05, 0.20) | 0.228 | 0.433 |
| mEOHP | 0.06 (-0.01, 0.12) | 0.082 | 0.260 | 0.10 (-0.01, 0.20) | 0.066 | 0.180 | 0.09 (-0.02, 0.21) | 0.119 | 0.365 | 0.08 (-0.05, 0.21) | 0.212 | 0.433 |
| MEHP | 0.03 (-0.03, 0.08) | 0.166 | 0.301 | 0.09 (0.02, 0.16)* | 0.017 | 0.093 | 0.05 (-0.03, 0.13) | 0.223 | 0.365 | 0.05 (-0.04, 0.14) | 0.246 | 0.433 |
| DNOP | 0.02 (-0.03, 0.08) | 0.369 | 0.528 | 0.02 (-0.06, 0.11) | 0.597 | 0.629 | 0.04 (-0.05, 0.14) | 0.370 | 0.462 | 0.00 (-0.10, 0.11) | 0.970 | 0.970 |
| mCPP | 0.02 (-0.03, 0.08) | 0.369 | 0.528 | 0.02 (-0.06, 0.11) | 0.597 | 0.629 | 0.04 (-0.05, 0.14) | 0.370 | 0.462 | 0.00 (-0.10, 0.11) | 0.970 | 0.970 |
| mBzP | -0.01 (-0.03, 0.02) | 0.622 | 0.778 | 0.01 (-0.03, 0.06) | 0.531 | 0.625 | -0.04 (-0.09, 0.01) | 0.130 | 0.365 | -0.01 (-0.06, 0.05) | 0.799 | 0.940 |
| mCIOP | 0.02 (-0.03, 0.07) | 0.490 | 0.654 | -0.02 (-0.10, 0.07) | 0.667 | 0.667 | 0.07 (-0.02, 0.16) | 0.127 | 0.365 | 0.06 (-0.04, 0.16) | 0.260 | 0.433 |
| mCINP | 0.04 (-0.01, 0.08) | 0.128 | 0.260 | 0.09 (0.02, 0.17)* | 0.017 | 0.093 | 0.05 (-0.04, 0.14) | 0.247 | 0.365 | -0.02 (-0.11, 0.08) | 0.704 | 0.939 |
| Values are obtained from linear mixed-effect models and represent the SDS change in weight (95% confidence interval (CI)) between birth and 4 years per natural log increase in the chemical exposure. Outcome SDs are sex-specific z-scores for age according to World Health Organization growth charts. Model is adjusted for race, education, marital status, insurance, parity, body mass index, alcohol and tabacco use. Models include inverse probability of censoring weights based on baseline characteristics. BP total bipshenol; BPA bisphenol A; BPS bisphenol S; PA phthalic acid; LMW low molecular weight phthalate; mEP mono-ethyl phthalate; mnBP mono-n-butyl phthalate; mIBP mono-isobutyl phthalate; HMW high molecular weight phthalate; DEHP di-(2-ethylhexyl) phthalate; mECPP mono-(2-ethyl-5-carboxypentyl) phthalate; mCMHP mono-(2-carboxymethyl) phthalate; mEHHP mono-(2-ethyl-5-hydroxyhexyl) phthalate; mEOHP mono-2(ethyl-5-oxohexyl) phthalate; mEHP mono-(2-ethylhexyl) phthalate; DNOP di-*n*-octyl phthalate; mCPP mono-(3-carboxypropyl) phthalate; mBzP mono-benzyl phthalate; mCIOP mono-(carboxyisooctyl) phthalate; mCINP mono-(carboxyisononyl) phthalate. *Nominal significant p-value <0.05 | | | | | | | | | | | | |

| **Table S16.** Associations of the individual and grouped pregnancy-averaged metabolites with infant growth patterns, adjusted model (n = 646) | | | | |
| --- | --- | --- | --- | --- |
|  |  | **Trimester 1** | | |
| **Chemical** | **Infant growth pattern** | **OR (95% CI)** | **Nominal p-value** | **FDR P-value** |
| BP | Growth deceleration | 0.92 (0.71, 1.18) | 0.521 | 0.952 |
|  | Normal growth | Reference |  |  |
|  | Growth acceleration | 1.05 (0.86, 1.27) | 0.640 | 0.809 |
| BPA | Growth deceleration | 0.91 (0.73, 1.12) | 0.370 | 0.943 |
|  | Normal growth | Reference |  |  |
|  | Growth acceleration | 1.01 (0.86, 1.18) | 0.913 | 0.926 |
| BPS | Growth deceleration | 0.99 (0.81, 1.20) | 0.909 | 0.952 |
|  | Normal growth | Reference |  |  |
|  | Growth acceleration | 0.97 (0.83, 1.13) | 0.724 | 0.809 |
| PA | Growth deceleration | 0.80 (0.67, 0.97) | 0.020* | 0.391 |
|  | Normal growth | Reference |  |  |
|  | Growth acceleration | 1.19 (1.02, 1.41) | 0.031 | 0.339 |
| LMW | Growth deceleration | 1.01 (0.80, 1.26) | 0.952 | 0.952 |
|  | Normal growth | Reference |  |  |
|  | Growth acceleration | 1.16 (0.98, 1.38) | 0.087 | 0.435 |
| mEP | Growth deceleration | 0.92 (0.76, 1.10) | 0.363 | 0.943 |
|  | Normal growth | Reference |  |  |
|  | Growth acceleration | 1.16 (1.01, 1.33) | 0.041* | 0.339 |
| mBP | Growth deceleration | 1.16 (0.92, 1.46) | 0.203 | 0.943 |
|  | Normal growth | Reference |  |  |
|  | Growth acceleration | 0.92 (0.77, 1.09) | 0.318 | 0.538 |
| mIBP | Growth deceleration | 1.11 (0.89, 1.40) | 0.377 | 0.943 |
|  | Normal growth | Reference |  |  |
|  | Growth acceleration | 1.03 (0.87, 1.22) | 0.728 | 0.809 |
| HMW | Growth deceleration | 0.98 (0.74, 1.26) | 0.863 | 0.952 |
|  | Normal growth | Reference |  |  |
|  | Growth acceleration | 1.11 (0.91, 1.35) | 0.310 | 0.538 |
| DEHP | Growth deceleration | 0.98 (0.74, 1.26) | 0.852 | 0.952 |
|  | Normal growth | Reference |  |  |
|  | Growth acceleration | 1.10 (0.90, 1.34) | 0.350 | 0.538 |
| mECPP | Growth deceleration | 0.96 (0.73, 1.25) | 0.781 | 0.952 |
|  | Normal growth | Reference |  |  |
|  | Growth acceleration | 1.14 (0.93, 1.40) | 0.196 | 0.538 |
| mCMHP | Growth deceleration | 0.91 (0.77, 1.07) | 0.259 | 0.943 |
|  | Normal growth | Reference |  |  |
|  | Growth acceleration | 1.14 (1.00, 1.30) | 0.051 | 0.339 |
| mEHHP | Growth deceleration | 0.91 (0.69, 1.18) | 0.491 | 0.952 |
|  | Normal growth | Reference |  |  |
|  | Growth acceleration | 1.05 (0.86, 1.28) | 0.604 | 0.809 |
| mEOHP | Growth deceleration | 0.93 (0.71, 1.20) | 0.596 | 0.952 |
|  | Normal growth | Reference |  |  |
|  | Growth acceleration | 1.10 (0.90, 1.33) | 0.348 | 0.538 |
| MEHP | Growth deceleration | 0.98 (0.82, 1.17) | 0.848 | 0.952 |
|  | Normal growth | Reference |  |  |
|  | Growth acceleration | 1.03 (0.90, 1.18) | 0.675 | 0.809 |
| DNOP | Growth deceleration | 0.99 (0.80, 1.21) | 0.904 | 0.952 |
|  | Normal growth | Reference |  |  |
|  | Growth acceleration | 1.12 (0.95, 1.32) | 0.166 | 0.538 |
| mCPP | Growth deceleration | 0.99 (0.80, 1.21) | 0.904 | 0.952 |
|  | Normal growth | Reference |  |  |
|  | Growth acceleration | 1.12 (0.95, 1.32) | 0.166 | 0.538 |
| mBzP | Growth deceleration | 0.93 (0.84, 1.03) | 0.147 | 0.943 |
|  | Normal growth | Reference |  |  |
|  | Growth acceleration | 1.05 (0.97, 1.15) | 0.245 | 0.538 |
| mCIOP | Growth deceleration | 1.13 (0.93, 1.38) | 0.216 | 0.943 |
|  | Normal growth | Reference |  |  |
|  | Growth acceleration | 1.01 (0.86, 1.18) | 0.926 | 0.926 |
| mCINP | Growth deceleration | 1.04 (0.85, 1.26) | 0.703 | 0.952 |
|  | Normal growth | Reference |  |  |
|  | Growth acceleration | 1.09 (0.94, 1.26) | 0.251 | 0.538 |
| Values are obtained from linear mixed-effect models and represent the odds ratio (OR) (95% confidence interval (CI)) on infant growth deceleration or acceleration between 0 and 2 years per natural log increase in the chemical exposure. Model is adjusted for race, education, marital status, insurance, parity, body mass index, alcohol and tabacco use. BP total bipshenol; BPA bisphenol A; BPS bisphenol S; PA phthalic acid; LMW low molecular weight phthalate; mEP mono-ethyl phthalate; mnBP mono-n-butyl phthalate; mIBP mono-isobutyl phthalate; HMW high molecular weight phthalate; DEHP di-(2-ethylhexyl) phthalate; mECPP mono-(2-ethyl-5-carboxypentyl) phthalate; mCMHP mono-(2-carboxymethyl) phthalate; mEHHP mono-(2-ethyl-5-hydroxyhexyl) phthalate; mEOHP mono-2(ethyl-5-oxohexyl) phthalate; mEHP mono-(2-ethylhexyl) phthalate; DNOP di-*n*-octyl phthalate; mCPP mono-(3-carboxypropyl) phthalate; mBzP mono-benzyl phthalate; mCIOP mono-(carboxyisooctyl) phthalate; mCINP mono-(carboxyisononyl) phthalate. FDR False Discovery Rate *Nominal significant p-value <0.05 | | | | |
